# Global Burden, Projection, and Inequalities Analysis of Cancer Attributable to Occupational Carcinogen Exposure in Individuals Aged Over 40 Years

**DOI:** 10.1002/cam4.71213

**Published:** 2025-09-04

**Authors:** Nanxi Hu, Hui Li, Kaili Yu, Yang Yu, Xiaohua Wu, Xinyi Huang, Hongtao Lin, Shuqing Zou, Jinluan Li

**Affiliations:** ^1^ Department of Quality Control Clinical Oncology School of Fujian Medical University, Fujian Cancer Hospital Fuzhou China; ^2^ Department of Radiation Oncology Clinical Oncology School of Fujian Medical University, Fujian Cancer Hospital Fuzhou China

**Keywords:** asbestos, cancer mortality, disability‐adjusted life years, Global Burden of Disease, lung cancer, occupational carcinogen

## Abstract

**Background:**

In this study, we investigated the global burden, projection, and inequalities of cancer attributable to occupational carcinogen exposure in individuals aged over 40 years.

**Methods:**

Using the Global Burden of Disease 2021 dataset, we examined age‐standardized disability‐adjusted life years (ASR‐DALYs) and deaths associated with cancer attributable to occupational carcinogen exposure. Statistical analyses included: the estimated Annual Percentage Change to assess trends (1990–2021); Bayesian age‐period‐cohort modeling for projections to 2030 and 2050; decomposition analysis to quantify contributions of aging, population growth, and epidemiological changes; and slope and concentration indices (SII, CI) to evaluate health inequalities by sociodemographic index (SDI).

**Results:**

In 2021, the global ASR‐DALYs attributable to occupational carcinogen exposure were 239.3 per 100,000 (95% uncertainty intervals [UI]: 180.76–300.91), with significant declines found since 1990. The ASR‐deaths in the same year were 11.45 per 100,000 (95% UI: 8.57–14.29). By 2050, ASR‐DALYs and ASR‐deaths are projected to decline to 177.24 and 8.50 per 100,000, respectively. Men exhibited higher DALYs and mortality (3.92 million DALYs, 0.18 million deaths) compared with women. From 1990 to 2021, high SDI regions exhibited the most substantial decline, whereas low‐middle SDI regions experienced the highest increase. The most prominent occupational carcinogens were asbestos (ASR‐DALYs: 142.36 per 100,000), silica (50.36 per 100,000), and diesel engine exhaust (20.56 per 100,000). Among the seven types of occupational cancers observe, tracheal, bronchial, and lung cancers exhibited the highest ASR‐DALY and ASR‐deaths. Population growth is the primary contributor to both DALYs and deaths globally, followed by epidemiological changes.

**Conclusion:**

This study highlights the declining global burden of cancer due to occupational exposure to carcinogens; however, significant disparities persist. Addressing occupational cancer risk in low‐SDI regions and under‐researched populations is crucial for reducing this health burden.

## 1 Introduction

The global aging workforce has heightened concerns about occupational hazards [1, 2]. Among occupational diseases, cancers due to occupational carcinogen exposure are a critical public health issue [3]. Preliminary all‐age population analyses confirm that people over 40 years of age have a higher risk of occupational cancer [4]. In addition, to reflect the latency issue (e.g., time‐lagged exposure windows), we limited our study population to individuals aged ≥ 40 years. Despite progress in workplace safety, the burden of occupational cancer and its associated mortality remains significant [5, 6].

Per the estimates of the International Labor Organization (ILO) and World Health Organization (WHO), approximately 5%–7% of the current global deaths are attributable to occupational exposure risks [7]. Other scholarly research reported over 35% of occupational exposure risk fatalities are attributed to cancer [8]. Per the Global Burden of Disease (GBD) Report in 2019, over 538,000 people die of cancer due to occupational exposure [9].

Cancers due to occupational carcinogen exposure risks are preventable because their etiological factors are well‐defined. Furthermore, the associated risks can be mitigated by reducing workers' exposure to carcinogenic agents [10, 11]. Numerous countries and regions have conducted localized analyses, [12, 13, 14, 15, 16] focusing on key risk factors, such as asbestos exposure [17] and UV radiation [18]. As a distinct population, workers aged ≥ 40 years typically have 20+ years of cumulative occupational exposure. However, the relative risk profiles of this demographic remain undercharacterized in occupational carcinogen research [19, 20, 21]. Additionally, a paucity of global data and trend analyses exists pertaining to cancer cases due to occupational exposure within this demographic region. Addressing this gap will enhance comprehension and development of mitigation strategies.

This study aimed to systematically evaluate the burdens, trends (1990–2021) and health inequality in mortality and disability‐adjusted life years (DALYs) from cancers attributed to occupational carcinogen exposure among individuals aged over 40 years, stratified by region, age, sex, and sociodemographic index (SDI). Furthermore, this study could forecast epidemiological trends and inform the development of globally targeted, enhanced health, and social security strategies grounded in academic research and theory.

## 2 Methods

### 2.1 Data Source and Study Population

This study uses data from the GBD 2021 database and Global Health Data Exchange (GHDx) results tool. GBD 2021 provided an extensive evaluation of health losses associated with 369 diseases, injuries, and impairments, considering 88 risk factors across 204 nations and territories. It was constructed using the most recent epidemiological data coupled with refined standardized methodologies to ensure accuracy and reliability [9]. The University of Washington's Institutional Review Board waived the necessity for informed consent to access GBD data [22]. This study complied with the Guidelines for Accurate and Transparent Health Estimates Reporting (GATHER) [23].

This study focused on the global burden of cancer attributed to occupational carcinogen exposure risks among individuals aged 40 years and older, including multiple dimensions such as global, 5 SDI regions, GBD‐defined super regions, sex, age intervals in a 5‐year segment, occupational carcinogen categories, and cancer types. The primary metrics included absolute numbers and age‐standardized rates (ASR) of DALYs and deaths. Occupational carcinogens included arsenic, asbestos, benzene, beryllium, cadmium, chromium, diesel engine exhaust, formaldehyde, nickel, polycyclic aromatic hydrocarbons, silica, sulfuric acid, and trichloroethylene [24]. The cancers under investigation included kidney, larynx, leukemia, mesothelioma, nasopharynx, ovarian, tracheal, bronchial, and lung cancer.

### 2.2 Age‐Standardization Approach

Age standardization was conducted to eliminate the impact of population age structure on overall rates, thereby enabling disease burden comparisons across different years and regions. Although GBD has provided ASR data, it covers the entire age span (from 0 to over 99 years). We calculated the ASR for age groups 40 years and above using the direct standardization method as follows. First, we identified the relevant age groups from the standard population composition that aligned with our research objectives. This selection was based on the age distribution data provided in the GBD database, focusing on groups that were 40 years and older. Second, we utilized the age group proportions from the GBD standard population 2021 [25, 26]; we constructed a new standard population tailored to our study's needs. This new standard population was designed to reflect the age distribution of the population of interest. Third, we applied the direct standardization method to calculate the ASR for our selected age groups. This process involved multiplying the crude rates of each age group by the proportion of that age group in the new standard population and summing these products to obtain the ASR.

### 2.3 Sociodemographic Index

The SDI is a composite measure that determines three dimensions of development: income (per capita gross domestic product), education (mean and expected years of schooling), and total fertility rate. A higher SDI, ranging between 0 and 1, indicates greater socioeconomic development [27]. The 204 countries and territories in the GBD 2021 were categorized into quintiles (low, low‐middle, middle, high‐middle, and high) according to the country‐level assessments of the SDI, providing a basis for comparing health outcomes across different levels of socioeconomic development.

### 2.4 Statistical Analysis

Numbers of DALYs and deaths with 95% uncertainty intervals (UIs), and their corresponding ASRs were the main indicators of cancer burden attributed to occupational carcinogen exposure risks. To assess the trends in ASR of DALYs and deaths from 1990 to 2021, we calculated the Estimated Annual Percentage Change (EAPC) [28]. The estimation of the EAPCs was conducted utilizing a linear regression model designed to depict the trajectory of ASR over a defined time span. The formula applied was as follows: *Y* = *α* + *βX* + *e*, with *Y* signifying the natural logarithm of the ASR, *X* representing the calendar year, *α* being the intercept, *β* indicating the slope or trend, and e standing for the error term. The EAPC was derived through the formula 100 × (exp(*β*) – 1), which reflects the annual percentage change. A linear regression model was employed to ascertain the 95% confidence intervals (CIs) associated with the EAPC. The average annual percentage changes (AAPC), calculated by a segmented linear regression model (such as the Joinpoint regression model), were also used for describing the trends in the ASR of DALYs and deaths. Joinpoint regression analysis was utilized to scrutinize temporal patterns in cancer burden attributed to occupational carcinogen exposure risk, pinpointing pivotal junctures at which substantial alterations took place. Regarding forecasting future trends, a Bayesian age‐period‐cohort (BAPC) model that incorporated integrated nested Laplace approximations was engaged [29, 30]. The computational procedures were carried out using the R package BAPC.

Pyramid charts were utilized to visualize the distribution of the ASR of DALYs and deaths, with the absolute numbers of DALYs and deaths associated with various occupational carcinogens and cancers across different age groups and sexes. Furthermore, heat maps were employed to depict the geographical distribution of the ASR for DALYs and deaths, along with the number of DALYs and deaths.

To further investigate the underlying factors' impact on cancer epidemiology attributed to occupational carcinogen exposure risk from 1990 to 2021, we utilized a decomposition analysis to decompose the DALYs and death changes in patients with cancer due to occupational carcinogen exposure risk owing to aging, population growth, and epidemiological changes. This approach allows the quantification of each factor's contribution to the overall change. Considering this decomposition analysis, we used the methods developed by Das Gupta, [31] which summarized the contribution of diverse elements to the observed variations by mathematically segregating the standardized impact of each contributing multiplicative factor.

In line with WHO guidelines, we employed the slope index of inequality (SII) and concentration index (CI) to assess disparities in cancer attributable to occupational carcinogen exposure across countries at various SDI levels [32]. The SII quantifies the inequality in DALYs and mortality rates across different SDI levels within a population. It was calculated by regressing the health outcome (e.g., ASR‐DALYs or ASR‐deaths) against the SDI ranking. The slope of the regression line represented the SII. A positive slope indicated that health outcomes improved as socioeconomic status increased, whereas a negative slope suggested the opposite. The CI was employed to evaluate the concentration of DALYs and deaths in different countries. Lorenz curves were constructed to represent the cumulative distribution of DALYs and deaths in relation to the cumulative proportion of the population ordered by SDI. The CI was calculated for each country by partitioning the region enclosed between the Lorenz curve and the line of perfect equality by the entire area beneath the line of perfect equality. CI values ranged between −1 and 1. A negative CI indicated a higher concentration of DALYs or deaths in populations with a lower SDI, whereas a positive CI suggested the opposite a—higher concentration in populations with higher SDI values.

All analyses were performed using R statistical computing software (version 4.4.1; Posit PBC, Boston, MA, US) and Stata MP17 (StataCorp LLC, College Station, TX, US). Visualizations were created using Adobe Illustrator 2023 (version 27.8.1; Adobe Inc., San Jose, CA, US) and Adobe Photoshop 2023 (version 24.7.0; Adobe Inc., San Jose, CA, US).

## 3 Results

### 3.1 Global Level

**FIGURE 1:**
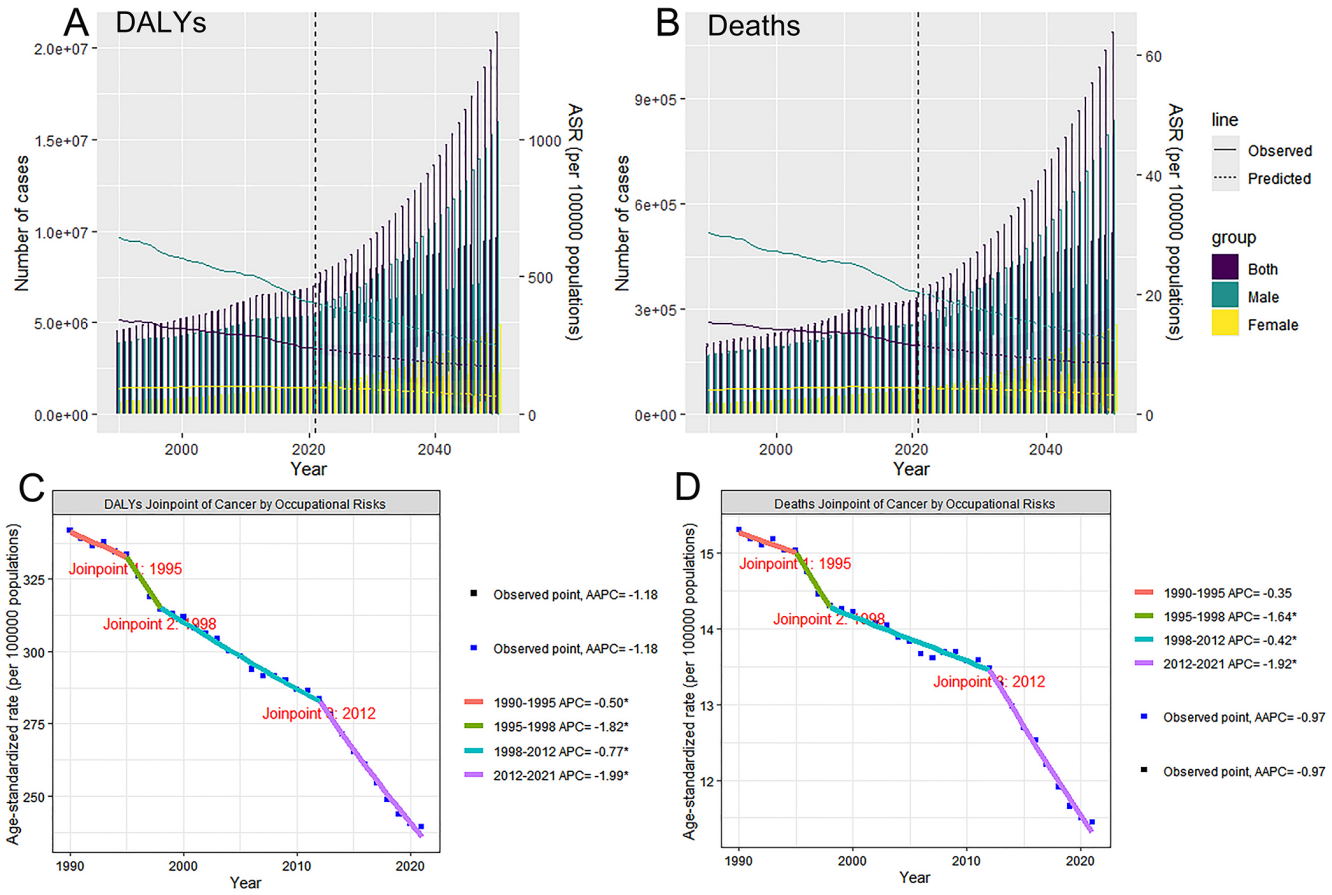
Trends and projections for the global burden of cancer attributable to occupational carcinogen exposure risks in individuals aged 40 and older. (A) DALYs. (B) Deaths. (C) Joinpoint regression analysis of DALYs. (D) Joinpoint regression analysis of Deaths. DALYs, disability‐adjusted life years.

In 2021, the global burden of cancer in individuals aged ≥ 40 years due to occupational carcinogen exposure remained substantial. The global DALYs reached 6,965,325.53 cases (95% UI: 5,270,229.28–8,760,723.68), with an ASR‐DALYs of 239.3 per 100,000 persons (95% UI: 180.76–300.91). The EAPC for ASR‐DALYs was −1.11 (95% CI: −1.19 to −1.04), indicating a consistent decrease in global DALYs. Significant alterations in the DALYs for cancers in individuals aged ≥ 40 years, attributable to occupational carcinogen exposure, were observed in the years 1995, 1998, and 2012. The most pronounced decline in DALYs was noted between 2012 and 2021, with an AAPC of −1.99. ASR‐DALYs are projected to decrease globally, declining to approximately 213.07 per 100,000 by 2030 and 177.24 per 100,000 by 2050, representing an approximately 25% decrease over the next two decades (Table 1, Figure 1).

**TABLE 1:** Number and age‐standardized rate (ASR) of disability‐adjusted life years (DALYs) for cancer in individuals aged 40 years and older attributable to occupational carcinogen exposure risks in 2021, with trends from 1990 to 2021 and projections for 2030 and 2050.

	2021		1990–2021
	DALYs cases	ASR‐DALYs per 100,000 (95% UI)	EAPC of DALYs
Global	6,965,325.53 (5,270,229.28 to 8,760,723.68)	239.3 (180.76 to 300.91)	−1.11 (−1.19 to −1.04)
Global Prediction of 2030	7,923,697.54 (6,275,545.71 to 9,571,849.38)	213.07 (168.55 to 257.58)	
Global Prediction of 2050	9,664,999.00 (130,578.77 to 20,898,838.97)	177.24 (2.03 to 393.99)	
Sex			
Male	5,445,234.13 (4,067,018.77 to 6,924,356.4)	405.57 (301.82 to 515.69)	−1.42 (−1.5 to −1.34)
Female	1,520,091.39 (1,075,001.07 to 1,990,144.4)	98.45 (69.64 to 128.9)	0.08 (0.01 to 0.14)
Age (years)			
40–44 years	149,084.6 (111,498.02 to 193,320.09)	29.8 (22.29 to 38.64)	−1.67 (−1.87 to −1.47)
45–49 years	303042.44 (216,757.77 to 403,193.1)	64 (45.78 to 85.15)	−0.94 (−1.12 to −0.76)
50–54 years	578,588.33 (421,502.04 to 761,125.57)	130.04 (94.74 to 171.07)	−1.34 (−1.5 to −1.17)
55–59 years	847,730.9 (629,531.46 to 1,090,016.05)	214.22 (159.08 to 275.45)	−1.52 (−1.64 to −1.39)
60–64 years	993,867.02 (768,349.82 to 1,271,935.86)	310.54 (240.07 to 397.42)	−1.47 (−1.59 to −1.35)
65–69 years	1,165,010.25 (910,303.64 to 1,461,709.09)	422.35 (330.01 to 529.91)	−1.56 (−1.6 to −1.51)
70–74 years	1,129,708.39 (889,371.38 to 1,370,594.9)	548.83 (432.07 to 665.86)	−1.2 (−1.29 to −1.12)
75–79 years	825,709.92 (635,467.05 to 998,292)	626.09 (481.84 to 756.94)	−0.83 (−0.96 to −0.71)
80–84 years	544,710.46 (397,145.79 to 670,159.86)	621.94 (453.45 to 765.17)	−0.28 (−0.49 to −0.07)
85–89 years	296,381.51 (204,114.46 to 370,994.25)	648.23 (446.43 to 811.42)	0.84 (0.64 to 1.04)
90–94 years	106,411.03 (71,353.14 to 135,774.23)	594.83 (398.86 to 758.97)	1.42 (1.26 to 1.58)
95+ years	25,080.68 (14,834.71 to 33,608.69)	460.17 (272.18 to 616.64)	1.71 (1.58 to 1.84)
SDI			
High	2,754,281.57 (2,083,324.58 to 3,381,584.78)	382.08 (289.26 to 470.62)	−1.6 (−1.71 to −1.49)
High‐middle	1,951,491.05 (1,431,853.26 to 2,566,071.46)	289.36 (211.92 to 380.86)	−0.94 (−1.03 to −0.85)
Middle	1,691,120.4 (1,217,842.93 to 2,276,308.18)	178.87 (128.44 to 241.01)	0.28 (0.18 to 0.37)
Low‐middle	459,590.65 (350,345.18 to 594,753.88)	89.57 (68.15 to 115.86)	0.98 (0.95 to 1.02)
Low	101,737.89 (71,376.91 to 139,098.82)	54.67 (38.13 to 75.26)	0.09 (−0.01 to 0.18)
GBD region			
High‐income	2,984,513.5 (2,251,476.85 to 3,661,340.54)	396.28 (299.43 to 487.86)	−1.66 (−1.77 to −1.56)
Southeast Asia, East Asia, and Oceania	2,360,322.52 (1,632,046.31 to 3,290,660.86)	235.04 (162 to 327.84)	0.66 (0.51 to 0.81)
South Asia	377,395.39 (281,505.41 to 495,925.63)	71.4 (53.2 to 93.84)	0.52 (0.45 to 0.58)
Central Europe, Eastern Europe, and Central Asia	518,410.81 (373,899.72 to 679,752.73)	235.18 (169.06 to 309.13)	−0.98 (−1.09 to −0.87)
North Africa and Middle East	295,865.15 (199,219.56 to 421,394.84)	187.88 (124.82 to 269.72)	−1.31 (−1.59 to −1.04)
Latin America and Caribbean	294,472.87 (230,040.94 to 367,428.31)	139.37 (108.63 to 174.02)	−0.55 (−0.63 to −0.48)
Sub‐Saharan Africa	134,345.28 (97,890.02 to 180,040.92)	79.87 (58.23 to 107.34)	−0.35 (−0.69 to 0)
Carcinogen risk			
Arsenic	291,718.86 (61,371.41 to 511,785.72)	9.79 (2.06 to 17.17)	−1.5 (−1.62 to −1.39)
Asbestos	4,077,098.8 (2,970,334.05 to 5,201,424.43)	142.36 (103.56 to 181.48)	−0.57 (−0.63 to −0.51)
Benzene	36,039.94 (10,772.31 to 59,650.42)	1.24 (0.37 to 2.05)	0.37 (0.29 to 0.46)
Beryllium	8740.42 (5002.15 to 13,016.33)	0.29 (0.17 to 0.44)	0.61 (0.53 to 0.69)
Cadmium	22,874.82 (13,776.67 to 33,010.07)	0.77 (0.46 to 1.11)	−0.58 (−0.63 to −0.54)
Chromium	49,507.79 (35,490.38 to 66,143.85)	1.66 (1.19 to 2.22)	−1.67 (−1.74 to −1.6)
Diesel engine exhaust	612,812.12 (425,736.19 to 824,757.01)	20.56 (14.28 to 27.67)	1.22 (1.14 to 1.3)
Formaldehyde	28803.52 (13815.91 to 50067.28)	0.99 (0.48 to 1.72)	−0.06 (−0.14 to 0.03)
Nickel	274,873.27 (24,229.64 to 709,843.78)	9.22 (0.81 to 23.81)	−0.96 (−1.07 to −0.86)
Polycyclic aromatic hydrocarbons	171,247.45 (107,200.81 to 242,876.37)	5.74 (3.6 to 8.14)	−0.75 (−0.78 to −0.72)
Silica	1,500,888.68 (666,479.78 to 2,374,194.42)	50.36 (22.36 to 79.66)	0.62 (0.57 to 0.67)
Sulfuric acid	111,342.13 (46,396.74 to 200,286.78)	3.74 (1.56 to 6.73)	−0.27 (−0.34 to −0.2)
Trichloroethylene	2375.01 (521.33 to 4380.79)	0.08 (0.02 to 0.15)	0.58 (0.51 to 0.66)
Cancer			
Kidney cancer	2375.01 (521.33 to 4380.79)	0.08 (0.02 to 0.15)	1.22 (1.14 to 1.3)
Larynx cancer	176,708.03 (98,551.68 to 279,818.63)	6.01 (3.35 to 9.52)	−1.94 (−2 to −1.88)
Leukemia	47,836.96 (22,129.51 to 72,839.01)	1.65 (0.76 to 2.51)	−0.21 (−0.29 to −0.14)
Mesothelioma	577,693.66 (515,278.53 to 636,504.36)	19.91 (17.73 to 21.94)	−0.46 (−0.59 to −0.34)
Nasopharynx cancer	16,858.09 (3450.9 to 37,449.47)	0.58 (0.12 to 1.29)	−1.53 (−1.72 to −1.34)
Ovarian cancer	97,940.95 (45,664.38 to 158,133.61)	3.44 (1.6 to 5.56)	−1.57 (−1.69 to −1.46)
Tracheal, bronchialus, and lung cancers	6,045,912.82 (4,392,432.56 to 7,806,077.89)	207.63 (150.61 to 267.98)	−1.14 (−1.21 to −1.06)

Mortality in the study population was estimated at 325,614.79 deaths (95% Ul: 244,681.75–406,667.12), with an age‐standardized death rate of 11.45 per 100,000 persons (95% UI: 8.57–14.29) and an EAPC of −0.85 (95% CI: −0.94 to −0.76). Parallel to the DALY trend, the mortality rate exhibited inflection points in 1995, 1998, and 2012. The period from 2012 to 2021 witnessed the most substantial reduction in mortality rates, with an AAPC of −1.92. By 2050, the mortality rate is projected to reach 8.50 per 100,000 (Table 1, Figure 1).

Sex disparities in the number of DALYs and deaths reached 3.92 million and 0.18 million, respectively, in 2021. Men aged ≥ 40 years exhibited a higher DALY and mortality rates compared with women globally. However, during the study period, both ASR‐DALYs and ASR‐deaths in men decreased, whereas those in women increased slightly. ASR‐DALYs and ASR‐deaths initially increased and then declined with advancing age, peaking in the 85–89 and 90–94 age groups, respectively. From 1990 to 2021, both indicators in individuals aged 40–84 years declined, whereas those aged > 85 years increased (Table 1).

### 3.2 Carcinogens and Cancer Type Level

**FIGURE 2:**
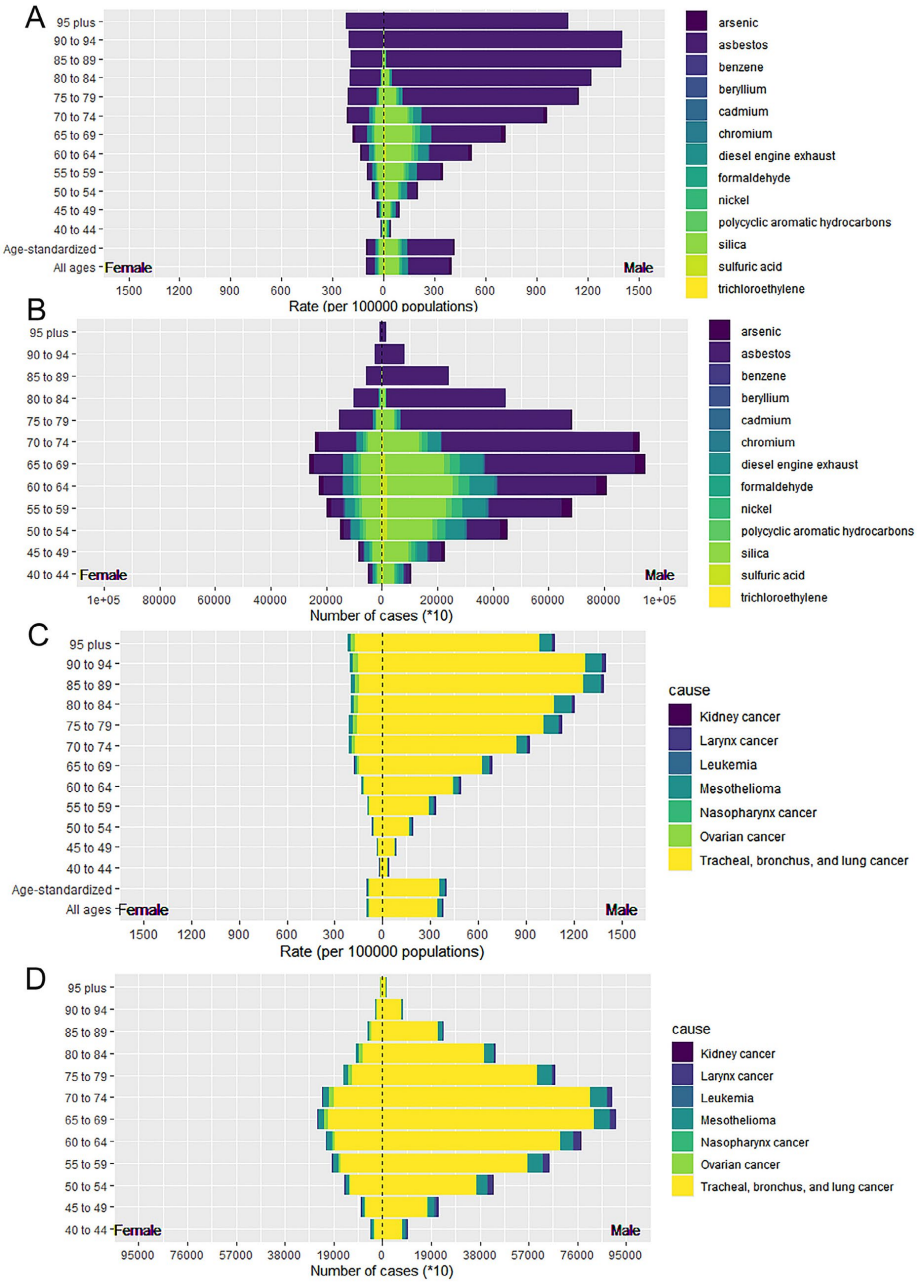
The burden of specific occupational carcinogens and cancer stratified by age and sex. (A) ASR‐DALYs for occupational carcinogen exposure. (B) Number of DALYs cases for occupational carcinogen exposure. (C) ASR‐DALYs for cancer attributable to occupational carcinogen exposure risks. (D) Number of DALYs cases for cancer attributable to occupational carcinogen exposure risks. ASR‐DALYs, age‐standardized disability‐adjusted life years.

Occupational carcinogen risks from asbestos, silica, and diesel engine exhaust were the most prominent contributors to ASR‐DALYs. Asbestos was the highest at 142.36 per 100,000 people (95% UI: 103.56–181.48), highlighting its substantial burden on public health. Silica and diesel engine exhaust had ASR‐DALYs of 50.36 per 100,000 (95% UI: 22.36–79.66) and 20.56 per 100,000 (95% UI: 14.28–27.67), respectively. Notably, although the burden of asbestos declined, silica and diesel engine exhaust emissions increased regarding ASR‐DALYs from 1990 to 2021. The EAPC for silica was 0.62 (95% UI: 0.57–0.67) and 1.22 (95% UI: 1.14–1.30) for diesel engine exhaust. Asbestos, silica, and diesel engine exhaust were also the top three contributors to ASR‐deaths among the occupational carcinogen risks (Table 1 and Table S1). ASR‐DALYs and ASR‐deaths attributable to occupational carcinogen risk varied significantly by sex and age group. The highest rates were observed in older age groups, with the 90–94 years age group experiencing the most considerable burden. These rates were significantly lower in women than in men across all age groups. Asbestos was the most prominent occupational carcinogen, and its impact increased with age, particularly in the male population. The impact of silica on ASR‐DALYs and ASR‐deaths initially increased with age and then decreased, reaching a peak in the age group of 65–69 years (Figure 2 and Figure S1).

Among the seven types of occupational cancers observed, tracheal, bronchial, and lung cancers exhibited a significantly higher ASR‐DALY rate than did the others, with a rate ten times greater than that of mesothelioma, which ranked second. The ASR‐DALY for tracheal, bronchus, and lung cancers was 207.63 per 100,000 population (95% UI: 150.61–267.98) and 19.91 per 100,000 (95% UI: 17.73–21.94) for mesothelioma. Tracheal, bronchial, and lung cancers exhibited a pronounced male predominance, with ASR‐DALYs in men aged 40–94 years increasing progressively with age, reaching over 1200 per 100,000 in the 85–89 age group. Similar trends were also found in ASR‐deaths (Table 1, Table S1, Figure 2, and Figure S1).

### 3.3 Regional Level

Regional disparities in the cancer burden attributable to occupational carcinogens were strongly associated with SDI levels in 2021. High‐SDI regions recorded the highest ASR‐DALYs (382.08 per 100,000; 95% UI: 289.26–470.62), whereas low‐SDI regions showed the lowest rates (54.67 per 100,000; 95% UI: 38.13–75.26). Notably, high‐SDI regions demonstrated the steepest decline in ASR‐DALYs from 1990 to 2021 (EAPC: −1.6; 95% CI: −1.71 to −1.49), whereas low‐middle‐SDI regions had the largest increase (EAPC: 0.98; 95% CI: 0.95–1.02). Similar stratification was observed for mortality rates, with high‐SDI regions reporting the highest ASR‐deaths and low‐SDI regions the lowest. Declining trends were exclusive to high and high‐middle SDI regions (Table 1 and Table S1).

**FIGURE 3:**
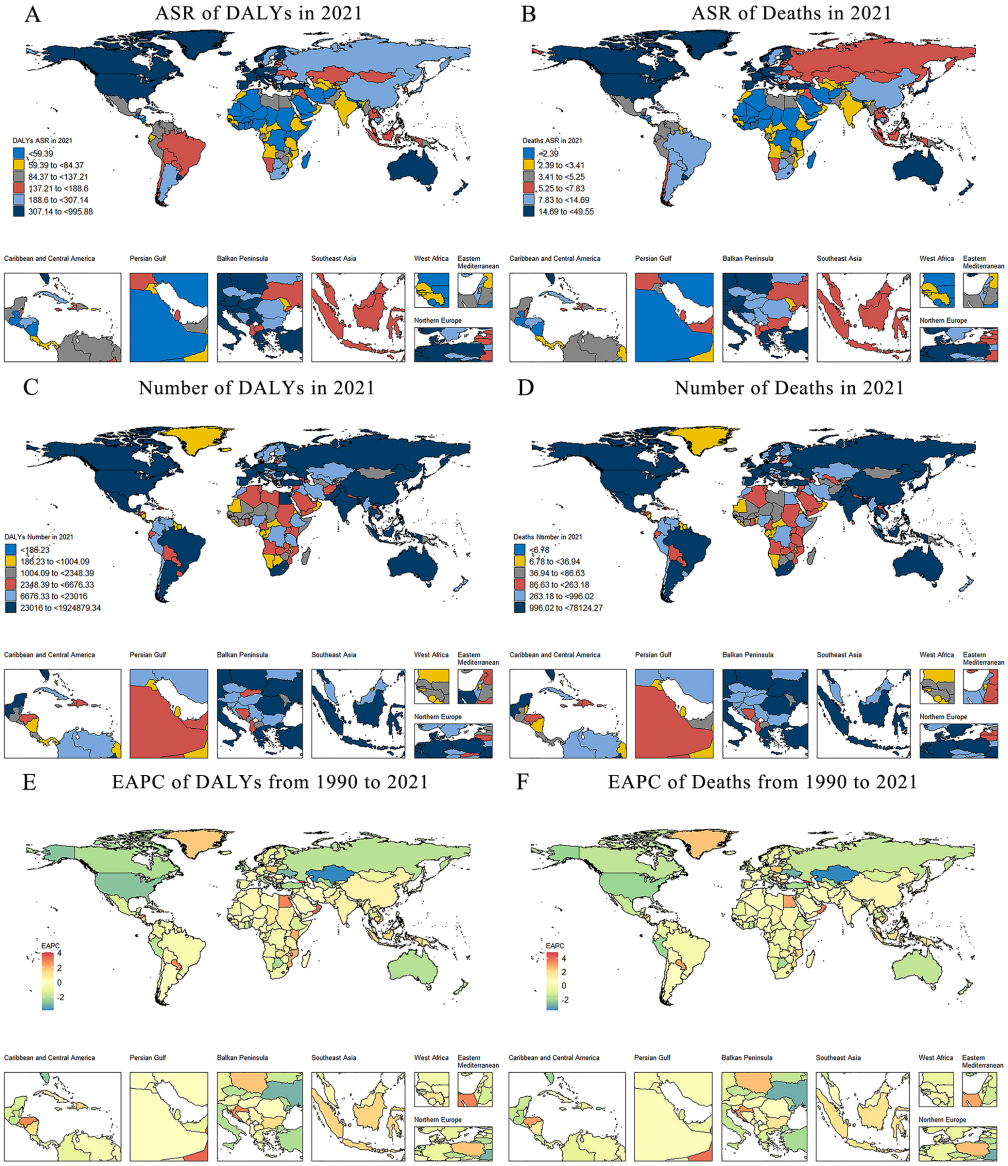
Global heatmap of the burden of cancer attributable to occupational carcinogen exposure risks in 2021 and changing trends from 1990 to 2021. (A) ASR of DALYs. (B) ASR of Deaths. (C) Number of DALYs. (D) Number of Deaths. (E) EAPC of DALYs. (F) EAPC of Deaths. ASR‐DALYs, age‐standardized disability‐adjusted life years; EAPC, estimated annual percentage change.

In 2021, the highest ASR‐DALYs among GBD super regions occurred in high‐income regions (396.28 per 100,000), followed by Central Europe, Eastern Europe, and Central Asia (235.18 per 100,000), and Southeast Asia, East Asia, and Oceania (235.04 per 100,000). Trend analysis revealed that five super regions experienced declining ASR‐DALYs from 1990 to 2021, with the most pronounced decrease in high‐income regions (EAPC: −1.66; 95% CI: −1.77 to −1.56). In contrast, only two regions showed increases: Southeast Asia, East Asia, and Oceania (EAPC: 0.66; 95% CI: 0.51–0.81) and South Asia (EAPC: 0.52; 95% CI: 0.45–0.58) (Table 1, Figure 3, Figure S2). Similar disparities were found in mortality rates. High‐income countries had the highest ASR‐deaths (20.17 per 100,000), followed by Southeast Asia, East Asia, and Oceania (9.77 per 100,000) and Central/Eastern Europe (9.62 per 100,000). From 1990 to 2021, high‐income regions exhibited the most significant decline (EAPC: −1.22; 95% CI: −1.32 to −1.12), whereas Southeast Asia and South Asia showed increasing trends (EAPC: 1.02 and 0.62, respectively) (Table S1, Figure 3 and Figure S2).

Geographic analysis identified clear carcinogen‐specific hotspots: asbestos‐related burdens predominated in industrialized regions (North America, Europe, and Australia; Figure S4), diesel engine exhaust exposure clustered in rapidly developing economies (East and Southeast Asia; Figure S9), and silica exposure was elevated in China and select European countries (Figure S13). Consistent with these patterns, tracheal, bronchial, and lung cancer rates peaked in North America and Australia (Figure S22), likely reflecting historical occupational exposure.

### 3.4 Decomposition Analysis

**FIGURE 4:**
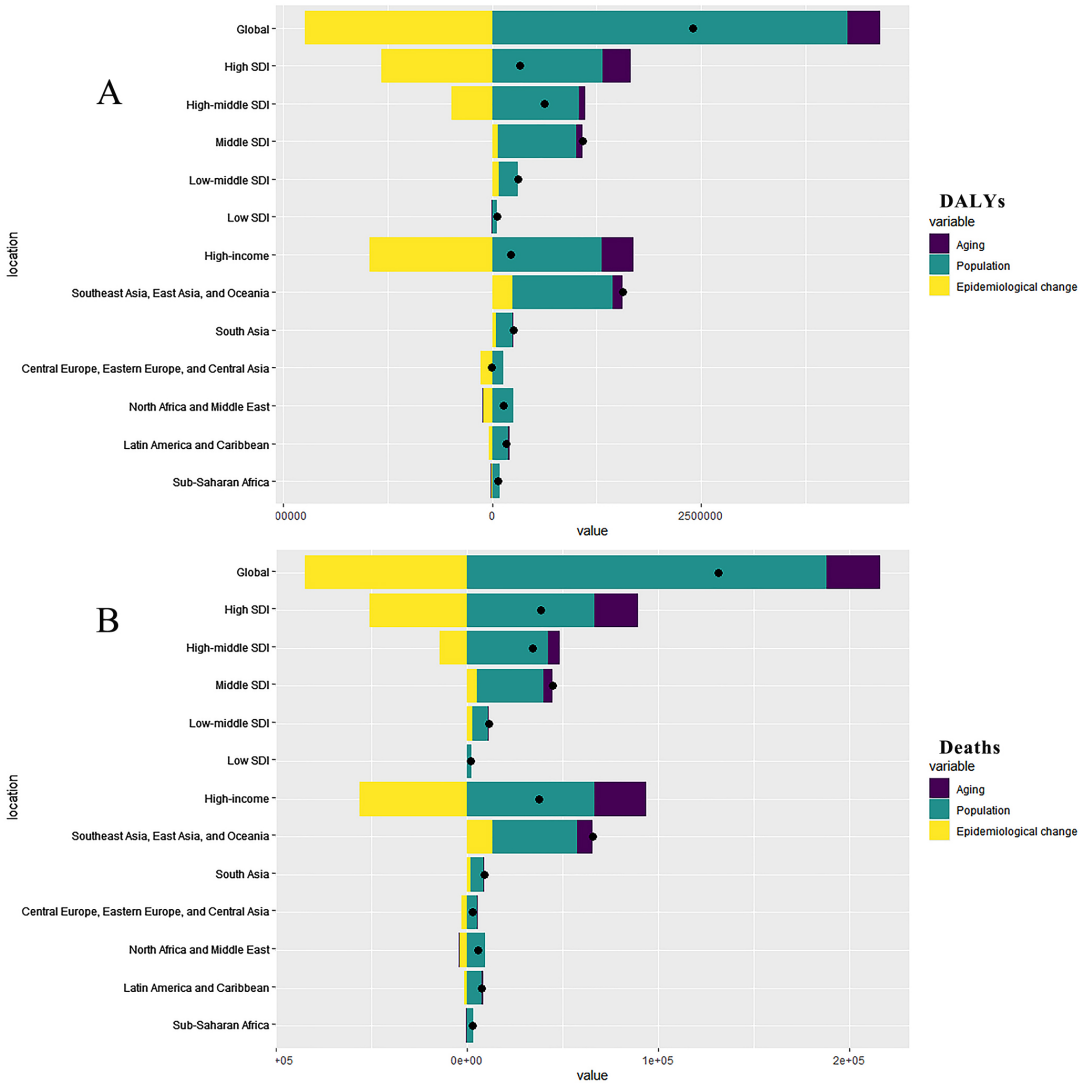
Global and regional decomposition analysis of the impact of occupational cancer epidemiology on (A) DALYs and (B) deaths. DALY, disability‐adjusted life years.

Decomposition analysis was conducted to assess how factors such as aging, population growth, and epidemiological changes could affect the epidemiology of cancer attributable to occupational carcinogen exposure. Globally, population growth is the primary contributor to both DALYs and deaths, followed by epidemiological changes; the impact of population aging is relatively minor. In most regions, particularly those with low and low‐middle SDI, population growth has emerged as the dominant driver of DALYs and deaths. The impact of epidemiological changes on DALYs and deaths was more significant in regions with a high SDI and income levels, attributable to the superior medical conditions and disease prevention strategies implemented in these areas (Table S2, Figure 4).

### 3.5 Health Inequalities Analysis

**FIGURE 5:**
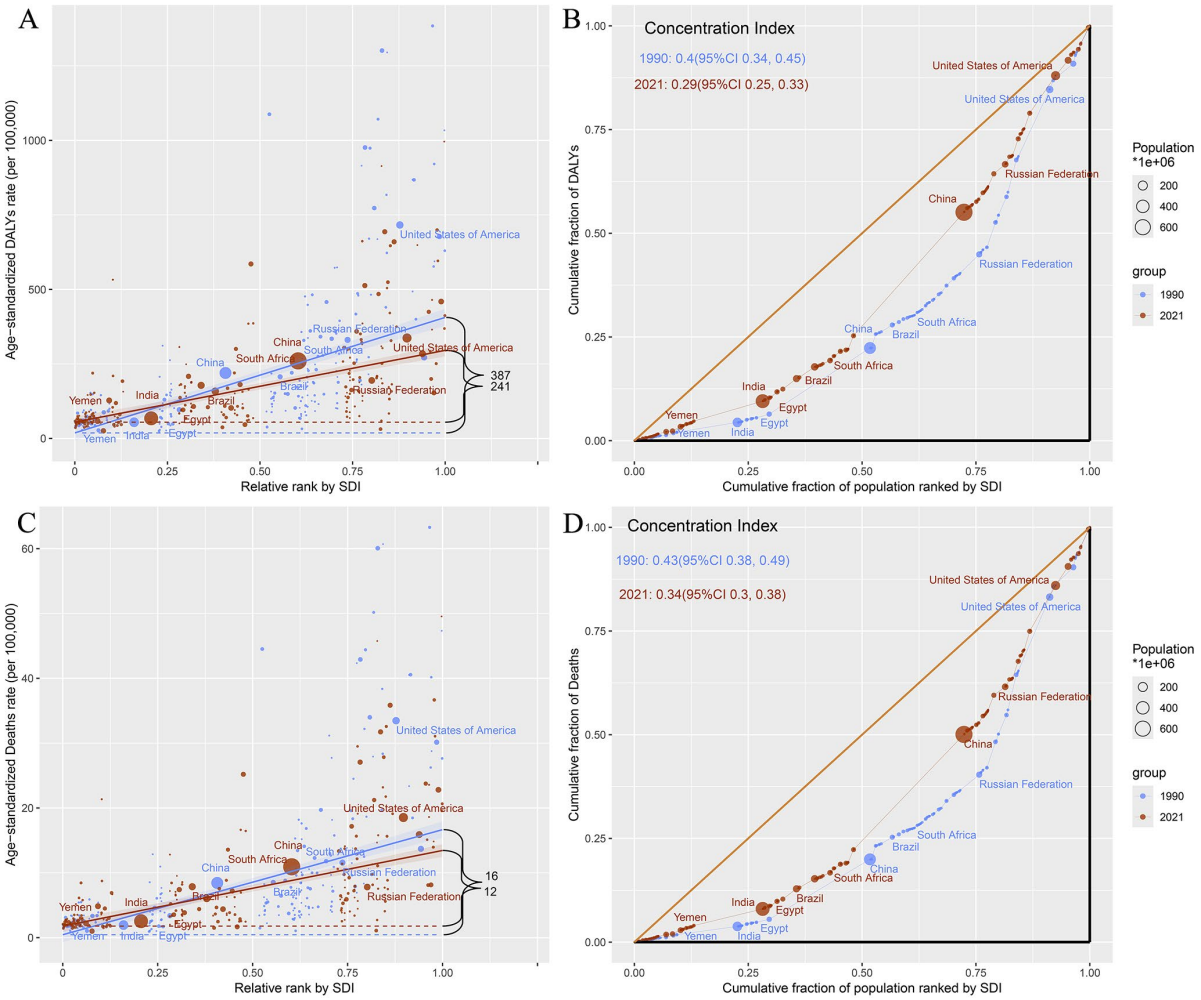
Health inequality of cancer attributable to occupational carcinogen exposure risks in 1990 and 2019. (A) Slope indices of inequality for DALYs. (B) Slope indices of inequality for deaths. (C) Concentration indices for DALYs. (D) Concentration indices for deaths. DALY, disability‐adjusted life years.

In 1990 and 2021, the SII (per 100,000 population) for DALYs was 387 and 241, respectively; the mortality rates were 16 and 12, respectively, indicating a negative correlation between SII and SDI. The marked reductions in the SII indicate a narrowing disparity in the age‐standardized cancer burden attributed to occupational carcinogen exposure between nations with higher and lower SDI levels over the observed period (Figure 5, Table S3). The Lorenz curve was used to display the distribution of DALYs and deaths across the global population when ranked by SDI. The CI for DALYs decreased from 0.4 in 1990 to 0.29 in 2021, and that for deaths decreased from 0.43 (1990) to 0.34 (2021). These changes indicate a shift towards a more equitable global distribution of DALYs and deaths. In both 1990 and 2021, the US showed a significant position on the graph, suggesting a high concentration of DALYs and deaths relative to its population size. China and India are depicted by larger circles, indicating substantial population sizes. The position of these countries below the line of equality suggests that they contribute less to the global burden of DALYs and deaths than their population sizes (Figure 5, Tables S4 and S5).

## 4 Discussion

Our results suggest that from 1990 to 2021, the global ASR‐DALYs and ASR‐deaths due to cancer attributable to occupational risk among individuals aged 40 years and older have shown a declining trend. This trend is consistent with the global burden pattern of occupational carcinogen‐related cancers across all age groups reported in previous studies [4, 33]. The reduction might have been largely influenced by global factors and national legal frameworks. Projections indicated this decline was likely to continue, with ASR‐DALYs and ASR‐deaths potentially decreasing by 20%–30% by 2050 if current policies are maintained and scaled. Prominent international organizations, including the ILO, United Nations (UN), Global Reporting Initiative (GRI), and International Organization for Standardization (ISO) emphasize the importance of worker's well‐being, risk prevention, and the establishment of safe working environments. By 2023, approximately all ILO member states have established authorities or bodies responsible for occupational safety and health (OSH), with 80% implementing legal provisions for workplace OSH committees to promote the fundamental right to a safe and healthy working environment [34]. However, emerging risks (e.g., novel occupational carcinogens, informal labor gaps) may slow progress in some regions. To sustain and enhance these advancements, strengthening regional cooperation, consolidating existing measures, and fostering safe and healthy global working environments is paramount.

High‐SDI regions had the highest ASR‐DALYs and ASR‐deaths, whereas low‐SDI regions had the lowest. However, high SDI regions exhibited the most substantial decline from 1990 to 2021. Conversely, low‐middle SDI regions experienced the highest increase. Cancer attributable to occupational risk diagnosis and registration systems is more comprehensive in developed countries with well‐established occupational disease surveillance systems, such as the Occupational Disease Surveillance System (ODSS), or cohort studies based on employed populations in multiple countries, such as the Nordic Occupational Cancer Study (NOCCA). In developing countries where exposure to occupational carcinogens may be higher, the cancer burden attributable to occupational risk is heavily underestimated [35]. Significant regional disparities exist in occupational safety and health (OSH) policies and programs. The proportion of member states with national OSH policies is significantly higher in high‐income (58%), upper‐middle‐income (45%), and lower‐middle‐income (47%) countries than in low‐income countries (26%). Approximately 50% of high‐income countries have established up‐to‐date national OSH programs, while the proportions are 25% and 21% for upper‐middle‐income and lower‐middle‐income countries, respectively. However, only 8% of low‐income countries have implemented such programs [34]. A narrowing disparity in the age‐standardized cancer burden is attributed to occupational risk between nations with higher and lower SDI levels; however, health inequality persists in lower SDI areas. Occupational exposure has not been adequately studied in developing countries owing to a lack of resources and government support [36]. A critical need arises for enhanced global commitment to advancing research on cancer attributable to occupational risk [37].

Globally, men aged ≥ 40 years exhibited a higher DALY and death rate for cancer attributable to occupational risk than did women. This disparity is primarily driven by two key factors. First, industries and occupations with elevated cancer risks, such as construction, metalworking, and mining, have historically been dominated by men [16]. Second, the risks faced by women in occupational settings are often underestimated. In many countries, women primarily engage in domestic work and the informal economy, which are largely invisible in public, economic, and institutional contexts. Consequently, work‐related accidents and diseases among women are often not recognized as occupational, remain uncompensated for by insurance systems, and are excluded from occupational health considerations [38].

With aging, the cancer burden escalates, particularly in those aged 65 and older, where a significant increase in DALYs and ASR‐DALYs is observed, signifying a higher cancer risk in this demographic. Many cancers attributable to occupational risk are diagnosed after retirement because cancer has a long preclinical phase [35]. For instance, malignant mesothelioma, linked to asbestos exposure, has a median latency period of approximately 40 years in various registries [39]. With the pace of population aging accelerating more rapidly than in previous decades, [40] the burden of occupational cancers in older adults is a growing challenge.

Asbestos is the most prominent carcinogen. The most prevalent asbestos‐related cancers are mesothelioma and lung cancer [41]. Asbestos use has a significant legacy of health and environmental consequences [42]. Many developed countries, such as those in the European Union [43], Canada [44], and the United Kingdom [45], have banned the import, export, sale, and use of asbestos. Over the past decade, a significant downward trend in mesothelioma incidence has been observed in some of these countries, [46] likely attributable to the ban on asbestos use. However, numerous developing countries continue to use asbestos, facing significant challenges. These include inadequate occupational cancer surveillance, aging populations, and limited research support. After asbestos, silica showed the next highest burden, followed by diesel engine exhaust. Notably, although the burden of asbestos declined, silica and diesel engine exhaust emissions increased. Epidemiological studies showed that high exposure to silica and diesel engine exhaust was significantly associated with cause‐specific mortality, particularly lung cancer [47, 48, 49, 50]. Given their substantial health burden and rising emissions, stricter occupational exposure limits and targeted interventions for silica and diesel engine exhaust are urgently needed. Effective control measures—such as improved ventilation, personal protective equipment, and cleaner energy alternatives—could significantly reduce preventable cancer deaths, particularly in high‐risk industries.

This study presents a novel longitudinal assessment of the cancer burden attributable to occupational carcinogen exposure among individuals aged 40 years and older from 1990 to 2021. It systematically analyzes secular trends in mortality, DALYs, and health inequalities associated with occupational cancer, while also projecting the disease burden over the next two decades. The findings offer critical insights into the evolving health impacts of occupational exposure and highlight the urgency for targeted preventive strategies and evidence‐based public health policies to mitigate the anticipated rise in cancer incidence. However, certain limitations should be acknowledged. The data quality within the GBD study varies across countries, potentially introducing bias into disease burden estimates, particularly in low‐ and middle‐income countries with weaker health data systems where information is often incomplete or unreliable [8]. Despite the use of advanced statistical models, these models rely on assumptions that may not fully capture the diverse demographic characteristics and epidemiological profiles of different countries [51]. The accurate assessment of certain occupational risks, such as diesel engine exhaust, [52] poses challenges, potentially leading to difficulties in accurately registering cancers attributable to occupational risk. Moreover, among the 47 agents classified as known occupational carcinogens by the International Agency for Research on Cancer (IARC) in 2017, only 13 are currently included in the GBD 2021 dataset [53]. Future studies should incorporate various databases, including the ODSS and NOCCA, to refine the accuracy of disease burden estimation.

To address the challenges faced by low‐SDI countries, practical measures may include: (1) strengthening occupational cancer surveillance through registries and digital reporting tools; (2) enforcing stricter regulations on high‐risk carcinogens such as asbestos and silica, aligned with international standards; (3) training healthcare workers and raising awareness among employers and workers; (4) fostering international collaborations for funding and technical support; and (5) extending protections to informal sectors. These steps can mitigate disparities and reduce the occupational cancer burden in resource‐limited settings.

## 5 Conclusions

This study highlights the declining global burden of occupational cancer attributable to risk factors between 1990 and 2021, driven by strengthened legal frameworks and improved surveillance systems. However, significant regional disparities persist, particularly between high‐ and low‐SDI countries, with the underreporting of occupational cancer in developing nations. Sex‐ and age‐related inequalities remain critical, with both men and older populations facing higher risks. Asbestos exposure continues to be a leading cause of death, particularly in regions without an asbestos ban. Continued international efforts are essential for enhancing occupational health policies and data accuracy.

## Author Contributions

**Nanxi Hu:** conceptualization (equal), formal analysis (equal), methodology (equal), writing – original draft (equal), writing – review and editing (equal). **Hui Li:** conceptualization (equal), formal analysis (equal), methodology (equal), writing – review and editing (equal). **Kaili Yu:** conceptualization (equal), methodology (equal), writing – original draft (equal), writing – review and editing (equal). **Yang Yu:** methodology (equal). **Xiaohua Wu:** methodology (equal). **Xinyi Huang:** software (equal). **Hongtao Lin:** validation (equal). **Shuqing Zou:** software (equal). **Jinluan Li:** funding acquisition (equal), supervision (equal).

## Conflicts of Interest

The authors declare no conflicts of interest.

## Supporting information


**Figure S1:** The burden of specific occupational carcinogens and cancer stratified by age and sex. (A) ASR‐deaths for occupational carcinogens exposure. (B) Number of deaths cases for occupational carcinogens exposure. (C) ASR‐deaths for occupational cancers. (D) Number of deaths cases for occupational cancers.
**Figure S2:** The heatmap for the global burden of cancer attributable to occupational exposure risks in 1990. (A) ASR of DALYs. (B) ASR of deaths. (C) Number of DALYs. (D) Number of deaths.
**Figure S3:** The heatmap for the global burden of arsenic exposure in 2021. (A) ASR of DALYs. (B) ASR of deaths. (C) Number of DALYs. (D) Number of deaths.
**Figure S4:** The heatmap for the global burden of asbestos exposure in 2021. (A) ASR of DALYs. (B) ASR of deaths. (C) Number of DALYs. (D) Number of deaths.
**Figure S5:** The heatmap for the global burden of benzene exposure in 2021. (A) ASR of DALYs. (B) ASR of deaths. (C) Number of DALYs. (D) Number of deaths.
**Figure S6:** The heatmap for the global burden of beryllium exposure in 2021. (A) ASR of DALYs. (B) ASR of deaths. (C) Number of DALYs. (D) Number of deaths.
**Figure S7:** The heatmap for the global burden of cadmium exposure in 2021. (A) ASR of DALYs. (B) ASR of deaths. (C) Number of DALYs. (D) Number of deaths.
**Figure S8:** The heatmap for the global burden of chromium exposure in 2021. (A) ASR of DALYs. (B) ASR of deaths. (C) Number of DALYs. (D) Number of deaths.
**Figure S9:** The heatmap for the global burden of diesel engine exhaust exposure in 2021. (A) ASR of DALYs. (B) ASR of deaths. (C) Number of DALYs. (D) Number of deaths.
**Figure S10:** The heatmap for the global burden of formaldehyde exposure in 2021. (A) ASR of DALYs. (B) ASR of deaths. (C) Number of DALYs. (D) Number of deaths.
**Figure S11:** The heatmap for the global burden of nickel exposure in 2021. (A) ASR of DALYs. (B) ASR of deaths. (C) Number of DALYs. (D) Number of deaths.
**Figure S12:** The heatmap for the global burden of polycyclic aromatic hydrocarbons exposure in 2021. (A) ASR of DALYs. (B) ASR of deaths. (C) Number of DALYs. (D) Number of deaths.
**Figure S13:** The heatmap for the global burden of silica exposure in 2021. (A) ASR of DALYs. (B) ASR of deaths. (C) Number of DALYs. (D) Number of deaths.
**Figure S14:** The heatmap for the global burden of sulfuric acid exposure in 2021. (A) ASR of DALYs. (B) ASR of deaths. (C) Number of DALYs. (D) Number of deaths.
**Figure S15:** The heatmap for the global burden of trichloroethylene exposure in 2021. (A) ASR of DALYs. (B) ASR of deaths. (C) Number of DALYs. (D) Number of deaths.
**Figure S16:** The heatmap for the global burden of kidney cancer in 2021. (A) ASR of DALYs. (B) ASR of deaths. (C) Number of DALYs. (D) Number of deaths.
**Figure S17:** The heatmap for the global burden of larynx cancer in 2021. (A) ASR of DALYs. (B) ASR of deaths. (C) Number of DALYs. (D) Number of deaths.
**Figure S18:** The heatmap for the global burden of leukemia in 2021. (A) ASR of DALYs. (B) ASR of deaths. (C) Number of DALYs. (D) Number of deaths.
**Figure S19:** The heatmap for the global burden of mesothelioma in 2021. (A) ASR of DALYs. (B) ASR of deaths. (C) Number of DALYs. (D) Number of deaths.
**Figure S20:** The heatmap for the global burden of nasopharynx cancer in 2021. (A) ASR of DALYs. (B) ASR of deaths. (C) Number of DALYs. (D) Number of deaths.
**Figure S21:** The heatmap for the global burden of ovarian cancer in 2021. (A) ASR of DALYs. (B) ASR of deaths. (C) Number of DALYs. (D) Number of deaths.
**Figure S22:** The heatmap for the global burden of tracheal bronchialus and lung cancer in 2021. (A) ASR of DALYs. (B) ASR of deaths. (C) Number of DALYs. (D) Number of deaths.
**Table S1:** Number and ASR of deaths for cancer in individuals aged 40 years and older attributable to occupational carcinogen exposure risks in 2021, with trends from 1990 to 2021 and projections for 2030 and 2050.
**Table S2:** Decomposition of aging, population, and epidemiological change for cancer in individuals aged 40 years and older attributable to occupational carcinogen exposure risks from 1990 to 2021.
**Table S3:** Slope indices of ASRs for cancer in individuals aged 40 years and older attributable to occupational carcinogen exposure risks from 1990 to 2021.
**Table S4:** Intercept indices of ASR for cancer in individuals aged 40 years and older attributable to occupational carcinogen exposure risks from 1990 to 2021.
**Table S5:** Concentration indices of ASR for cancer in individuals aged 40 years and older attributable to occupational carcinogen exposure risks from 1990 to 2021.

## Data Availability

The data from this study can be accessed openly through the GBD 2021 online database, as outlined in the Section 2.
