# Supplementary material for: Global Burden, Projection, and Inequalities Analysis of Cancer Attributable to Occupational Carcinogen Exposure in Individuals Aged Over 40 Years
Source: Cancer Med. 2025 Sep 4;14(17):e71213. doi: 10.1002/cam4.71213 (PMC12409659; doi:10.1002/cam4.71213)
Supplement: Supplementary file 1 — Figure S1: The burden of specific occupational carcinogens and cancer stratified by age and sex. (A) ASR‐deaths for occupational carcinogens exposure. (B) Number of deaths cases for occupational carcinogens exposure. (C) ASR‐deaths for occupational cancers. (D) Number of deaths cases for occupational cancers. Figure S2: The heatmap for the global burden of cancer attributable to occupational exposure risks in 1990. (A) ASR of DALYs. (B) ASR of deaths. (C) Number of DALYs. (D) Number of deaths. Figure S3: The heatmap for the global burden of arsenic exposure in 2021. (A) ASR of DALYs. (B) ASR of deaths. (C) Number of DALYs. (D) Number of deaths. Figure S4: The heatmap for the global burden of asbestos exposure in 2021. (A) ASR of DALYs. (B) ASR of deaths. (C) Number of DALYs. (D) Number of deaths. Figure S5: The heatmap for the global burden of benzene exposure in 2021. (A) ASR of DALYs. (B) ASR of deaths. (C) Number of DALYs. (D) Number of deaths. Figure S6: The heatmap for the global burden of beryllium exposure in 2021. (A) ASR of DALYs. (B) ASR of deaths. (C) Number of DALYs. (D) Number of deaths. Figure S7: The heatmap for the global burden of cadmium exposure in 2021. (A) ASR of DALYs. (B) ASR of deaths. (C) Number of DALYs. (D) Number of deaths. Figure S8: The heatmap for the global burden of chromium exposure in 2021. (A) ASR of DALYs. (B) ASR of deaths. (C) Number of DALYs. (D) Number of deaths. Figure S9: The heatmap for the global burden of diesel engine exhaust exposure in 2021. (A) ASR of DALYs. (B) ASR of deaths. (C) Number of DALYs. (D) Number of deaths. Figure S10: The heatmap for the global burden of formaldehyde exposure in 2021. (A) ASR of DALYs. (B) ASR of deaths. (C) Number of DALYs. (D) Number of deaths. Figure S11: The heatmap for the global burden of nickel exposure in 2021. (A) ASR of DALYs. (B) ASR of deaths. (C) Number of DALYs. (D) Number of deaths. Figure S12: The heatmap for the global burden of polycyclic aromatic hydrocarbons exposure [file CAM4-14-e71213-s001.doc]

**Supplementary Material**

# Supplementary Figures and Tables

## Supplementary Figures


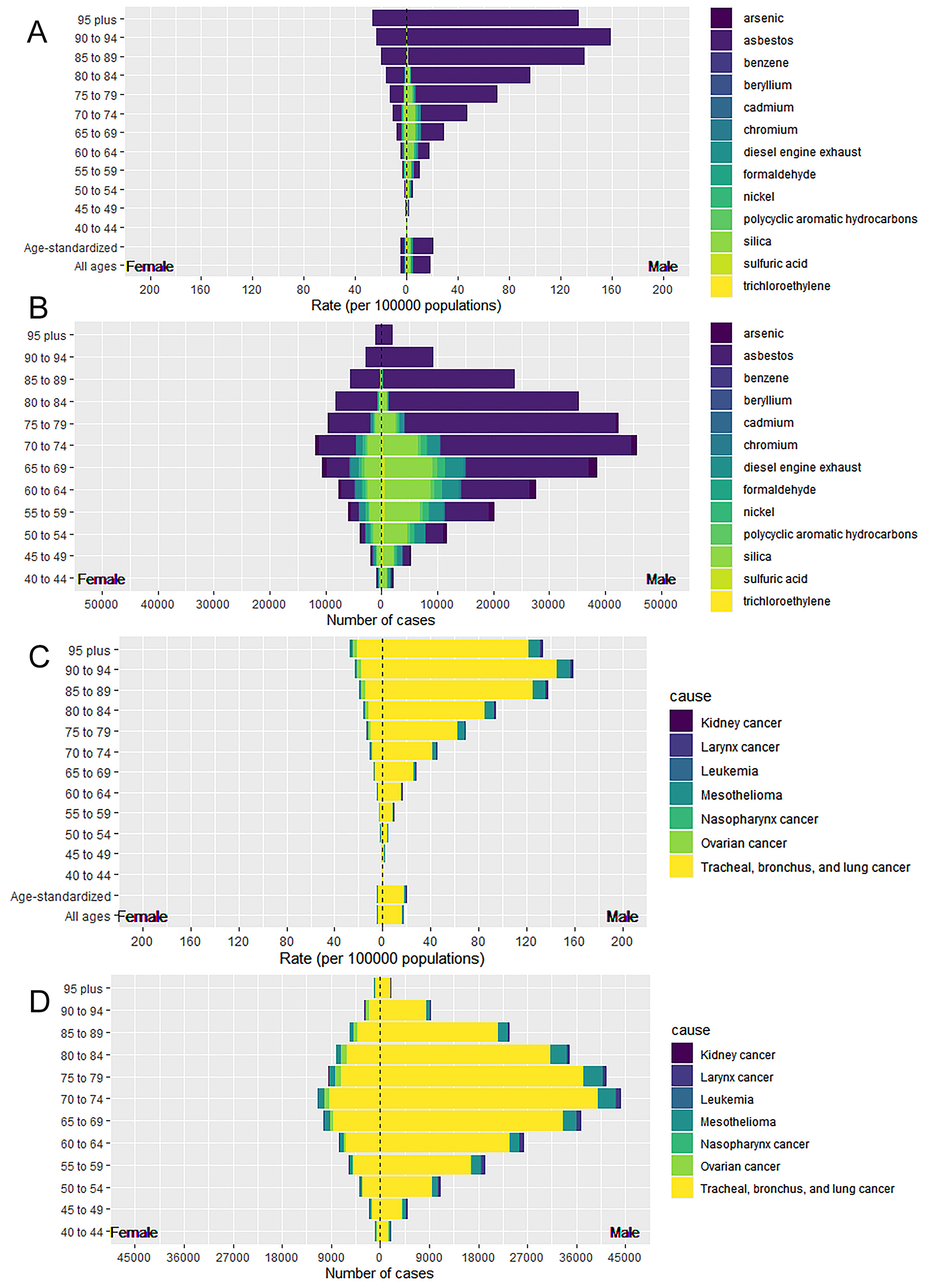


Figure S1 The burden of specific occupational carcinogens and cancer stratified by age and sex.(A) ASR-deaths for occupational carcinogens exposure. (B)Number of deaths cases for occupational carcinogens exposure. (C)ASR-deaths for occupational cancers. (D)Number of deaths cases for occupational cancers.


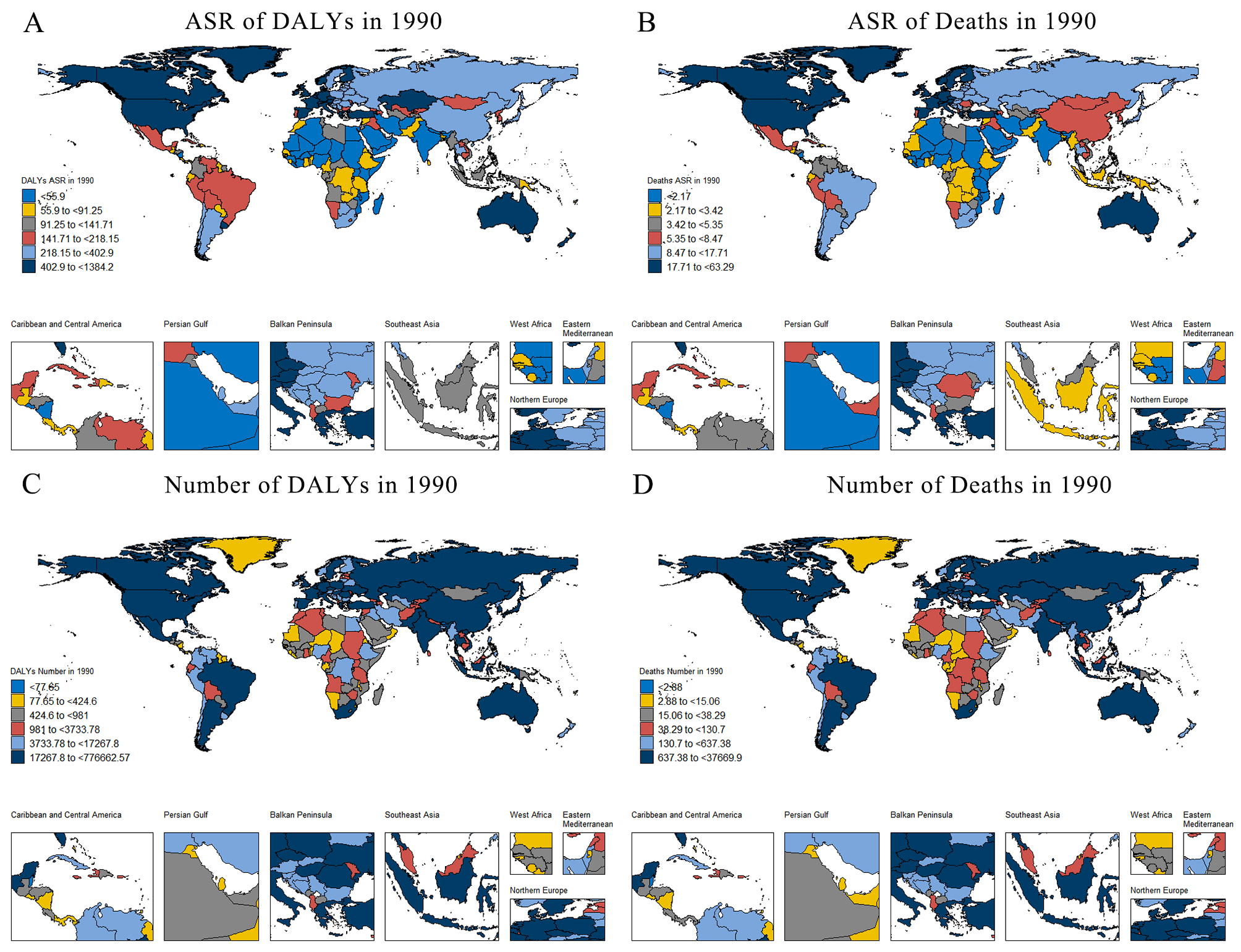


Figure S2 The heatmap for the global burden of cancer attributable to occupational exposure risks in 1990. (A) ASR of DALYs.(B) ASR of Deaths.(C)Number of DALYs.(D)Number of Deaths.


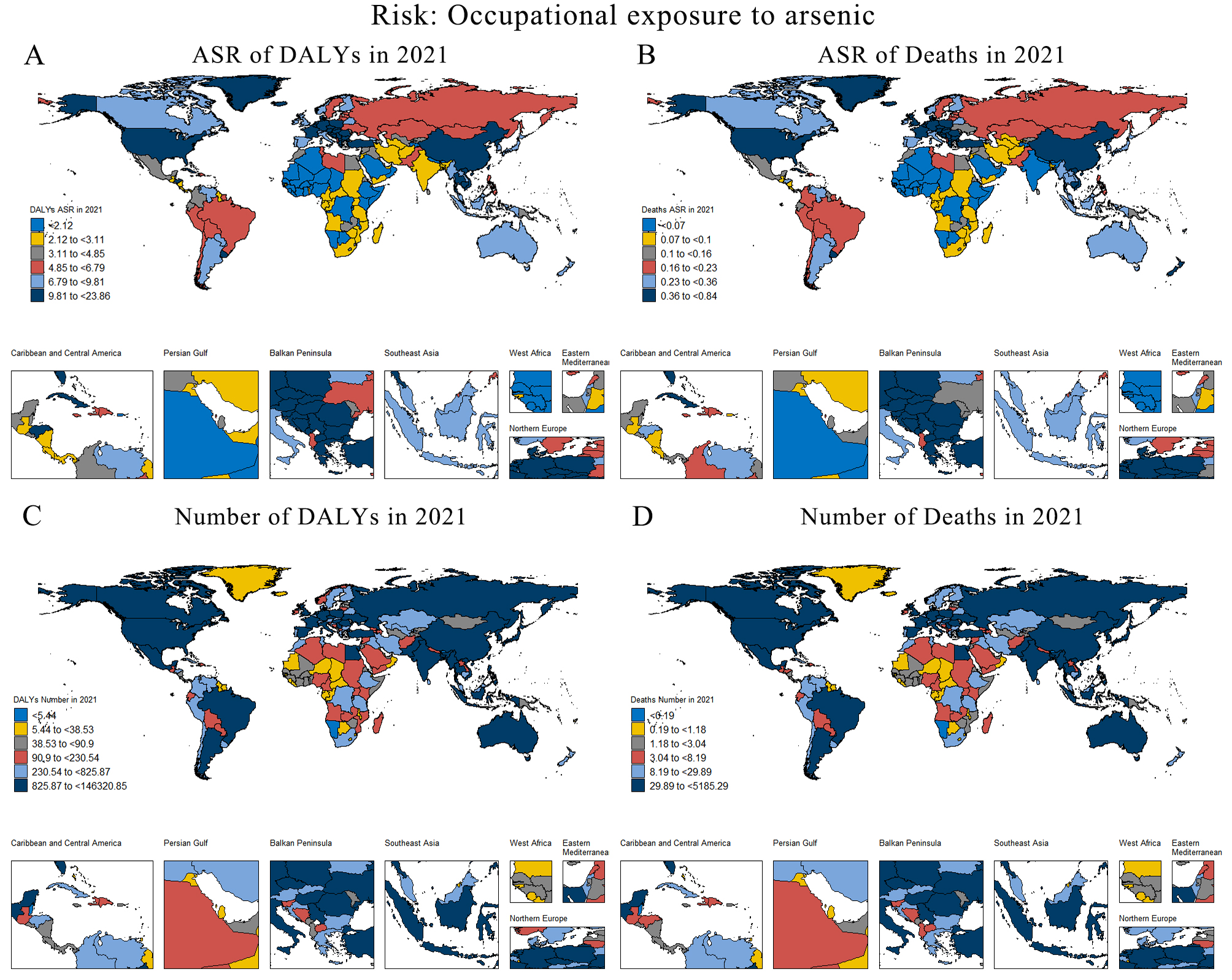


Figure S3 The heatmap for the global burden of arsenic exposure in 2021. (A) ASR of DALYs.(B) ASR of Deaths.(C)Number of DALYs.(D)Number of Deaths.


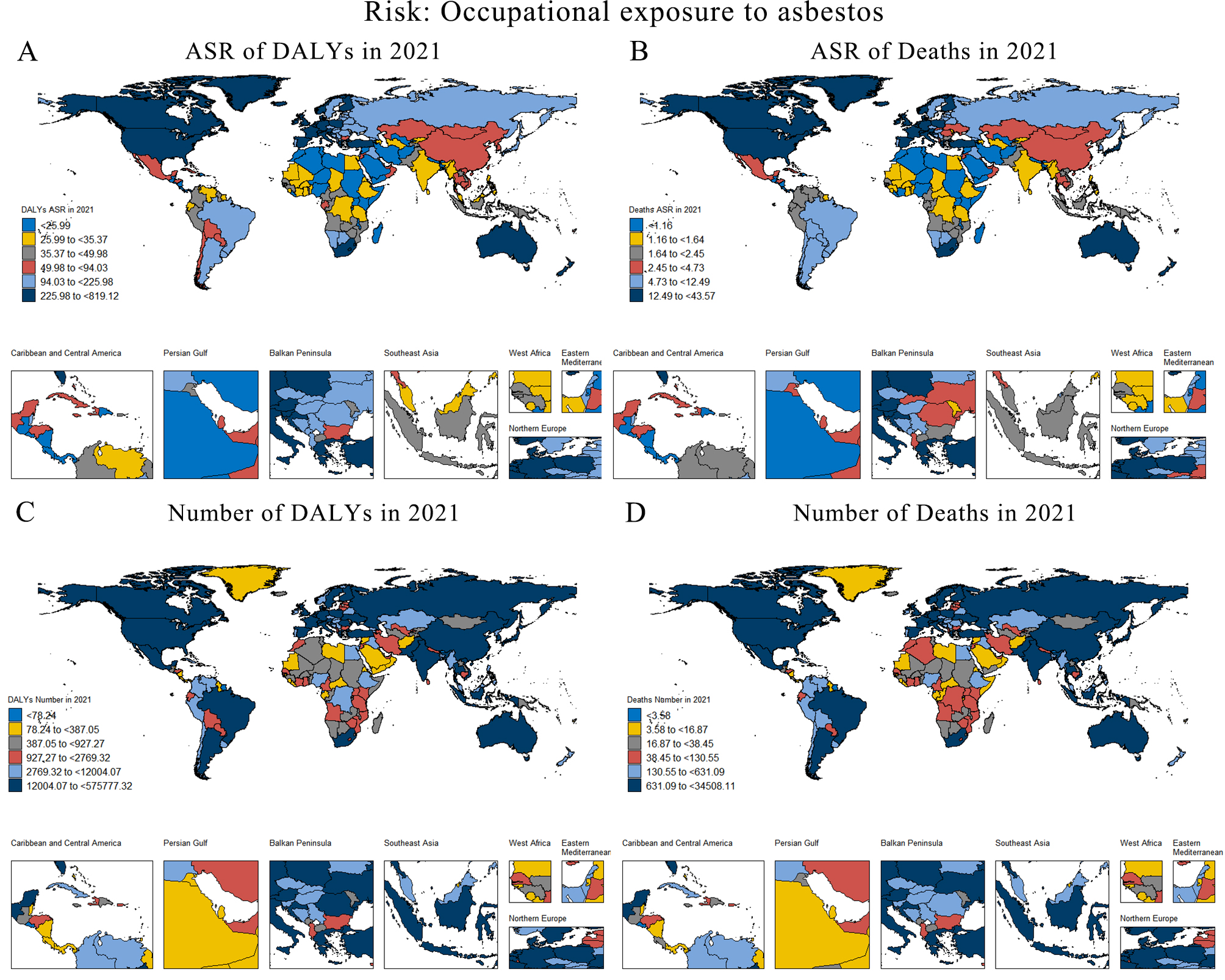


Figure S4 The heatmap for the global burden of asbestos exposure in 2021. (A) ASR of DALYs.(B) ASR of Deaths.(C)Number of DALYs.(D)Number of Deaths.


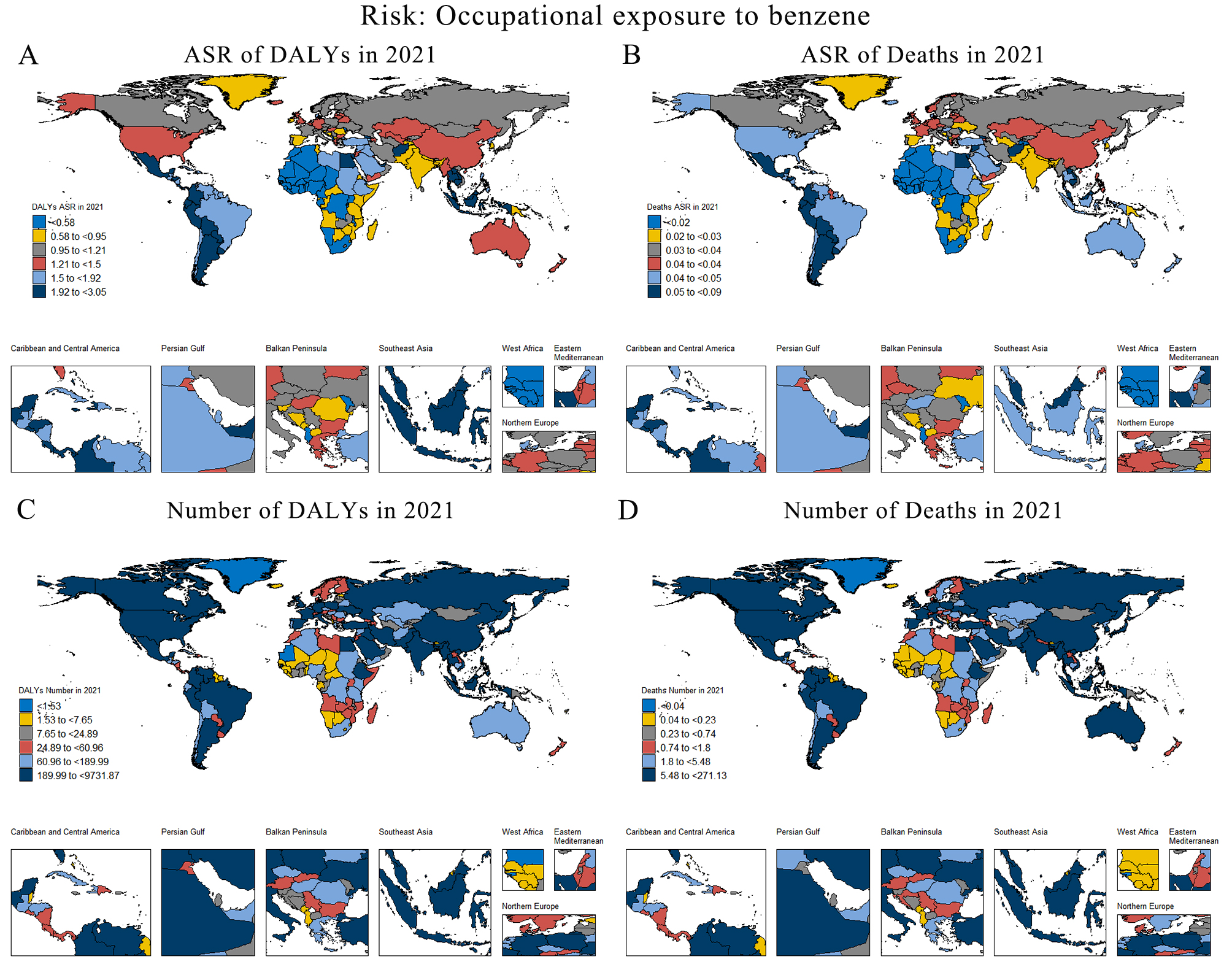


Figure S5 The heatmap for the global burden of benzene exposure in 2021. (A) ASR of DALYs.(B) ASR of Deaths.(C)Number of DALYs.(D)Number of Deaths.


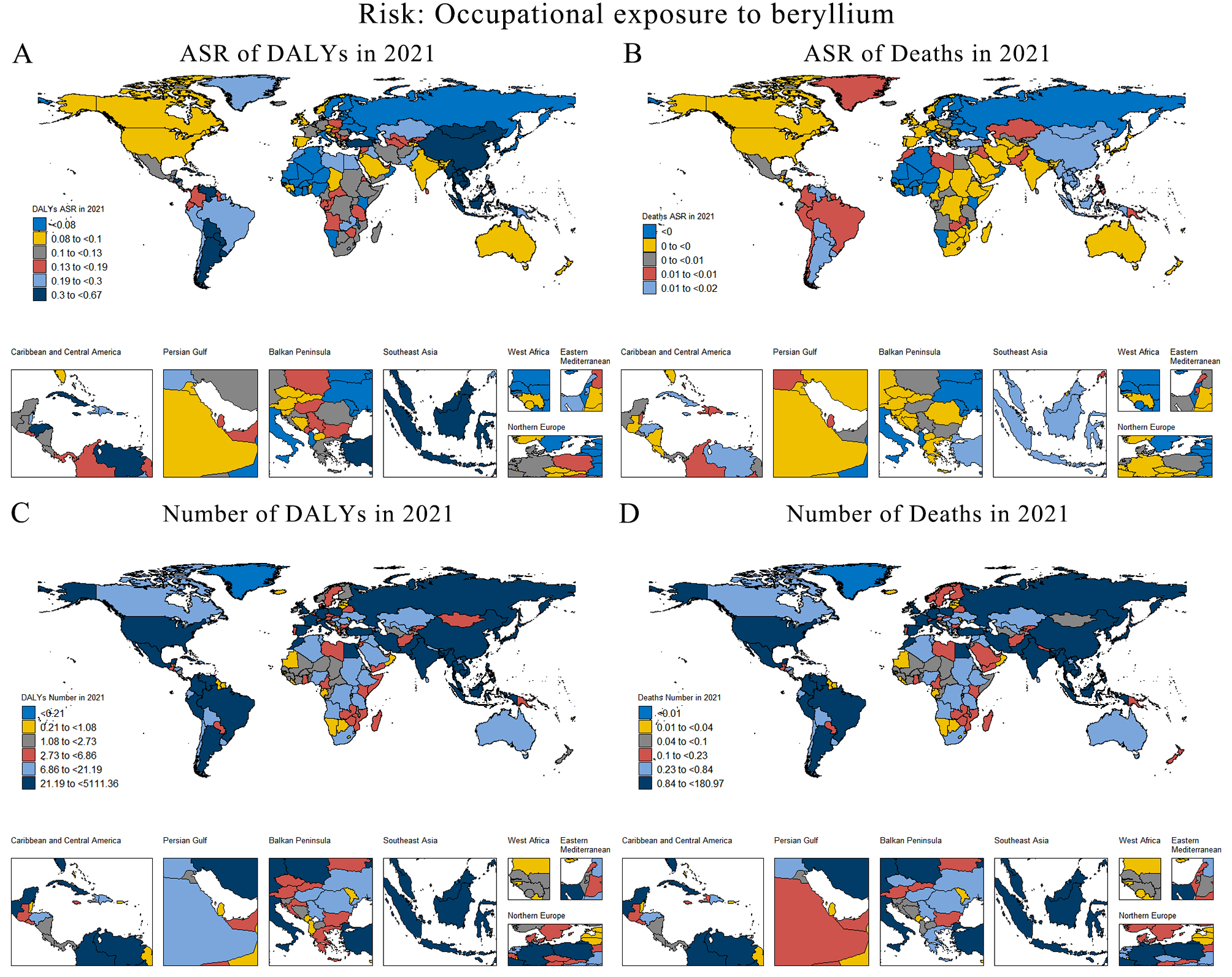


Figure S6 The heatmap for the global burden of beryllium exposure in 2021. (A) ASR of DALYs.(B) ASR of Deaths.(C)Number of DALYs.(D)Number of Deaths.


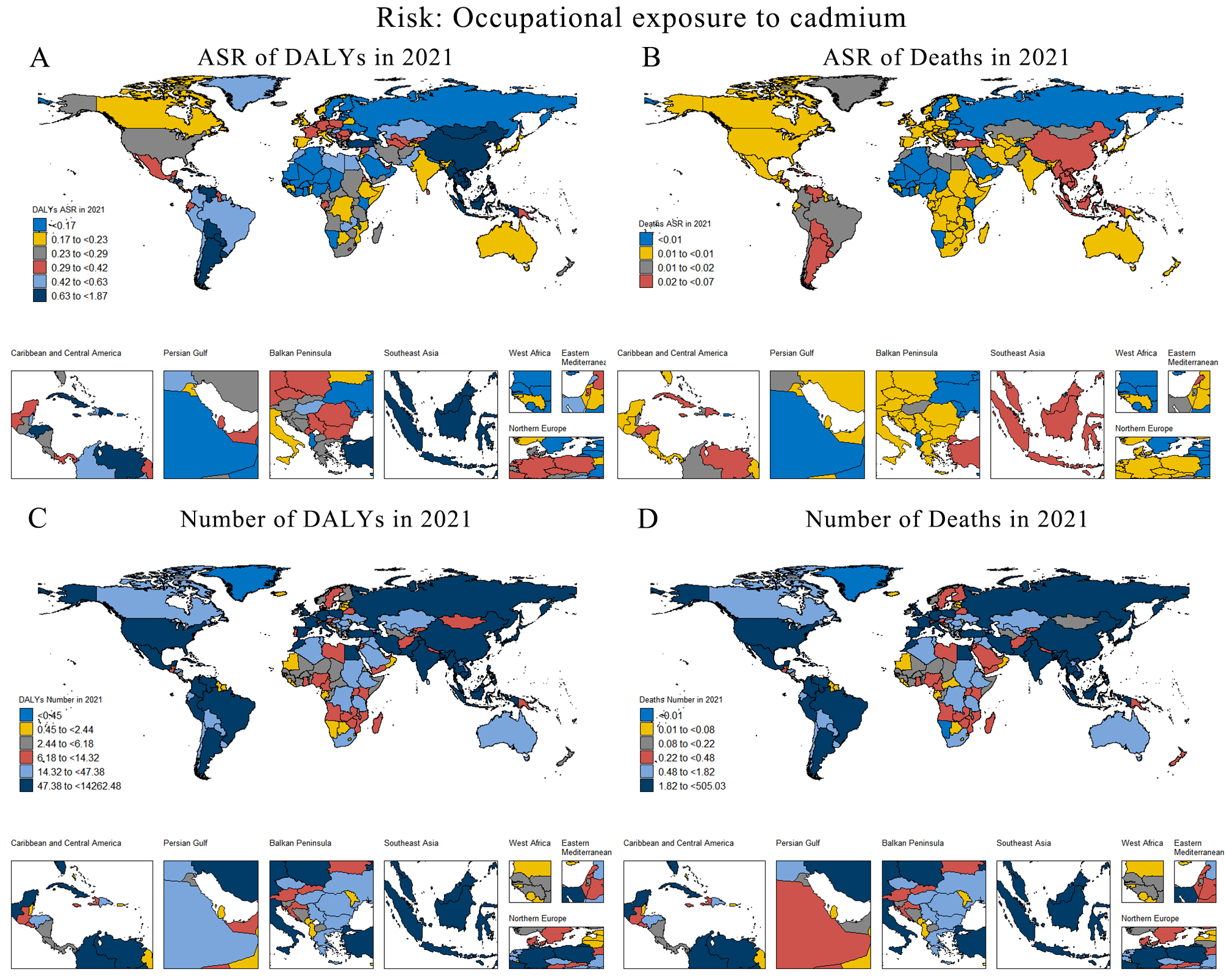


Figure S7 The heatmap for the global burden of cadmium exposure in 2021. (A) ASR of DALYs.(B) ASR of Deaths.(C)Number of DALYs.(D)Number of Deaths.


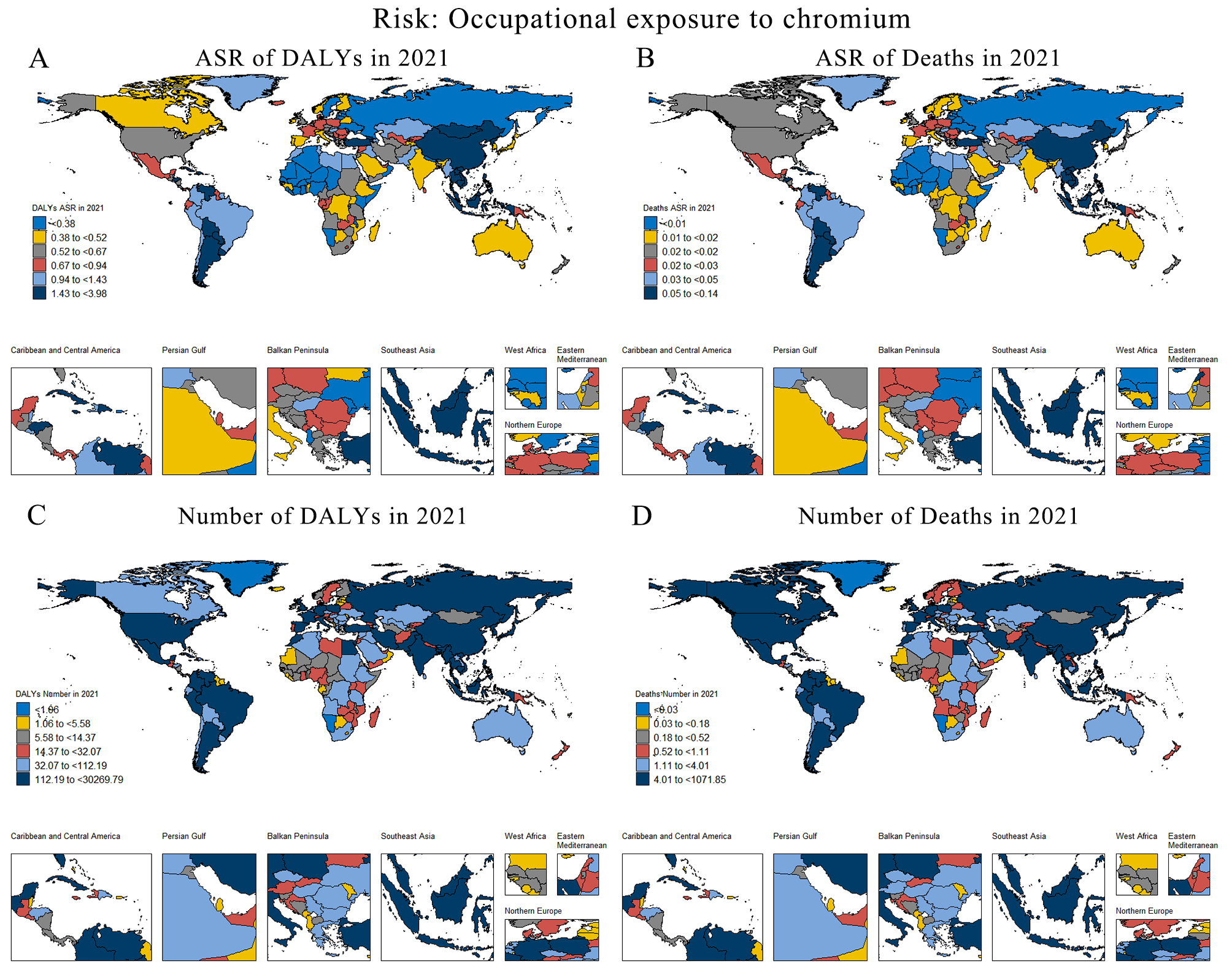


Figure S8 The heatmap for the global burden of chromium exposure in 2021. (A) ASR of DALYs.(B) ASR of Deaths.(C)Number of DALYs.(D)Number of Deaths.


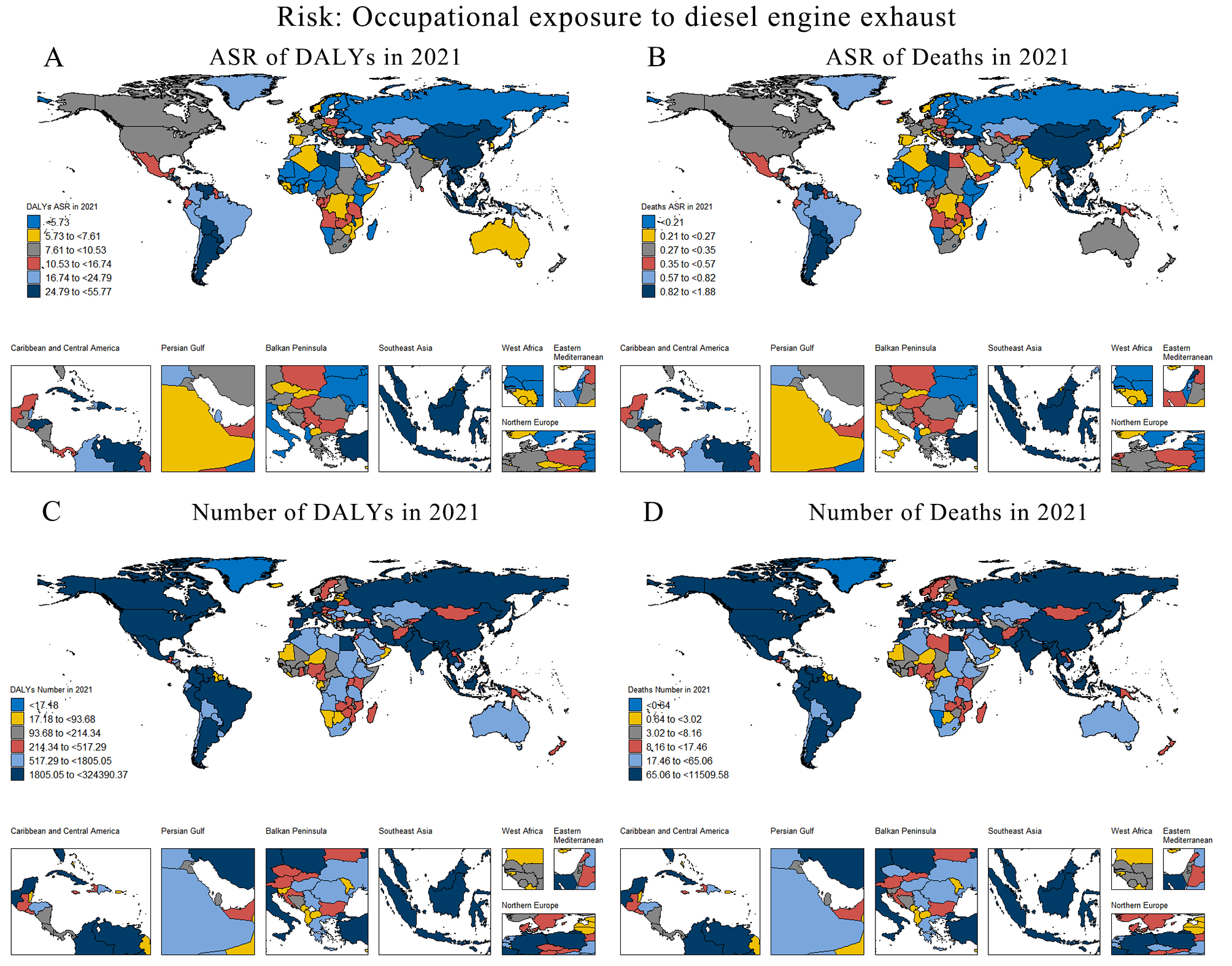


Figure S9 The heatmap for the global burden of diesel engine exhaust exposure in 2021. (A) ASR of DALYs.(B) ASR of Deaths.(C)Number of DALYs.(D)Number of Deaths.


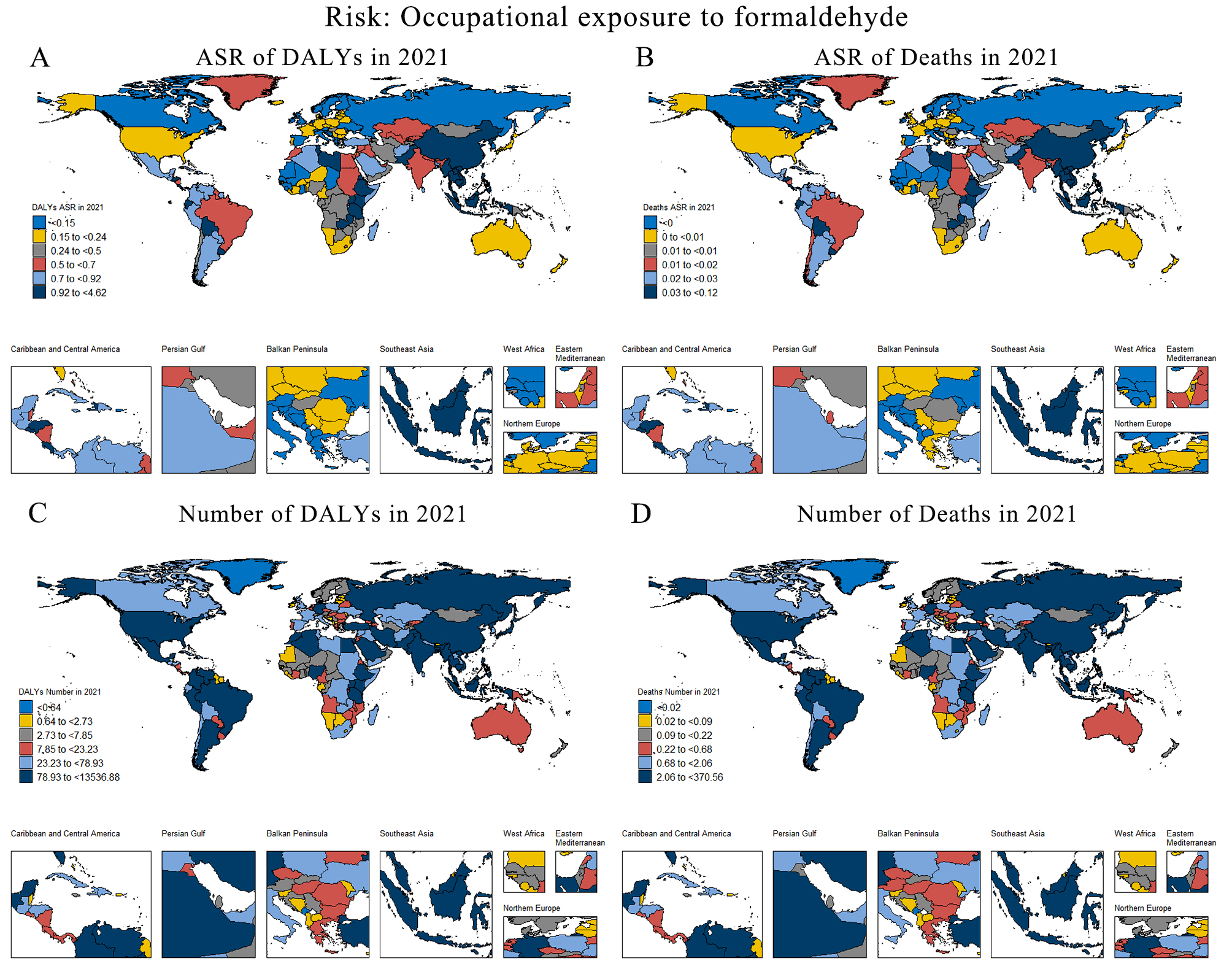


Figure S10 The heatmap for the global burden of formaldehyde exposure in 2021. (A) ASR of DALYs.(B) ASR of Deaths.(C)Number of DALYs.(D)Number of Deaths.


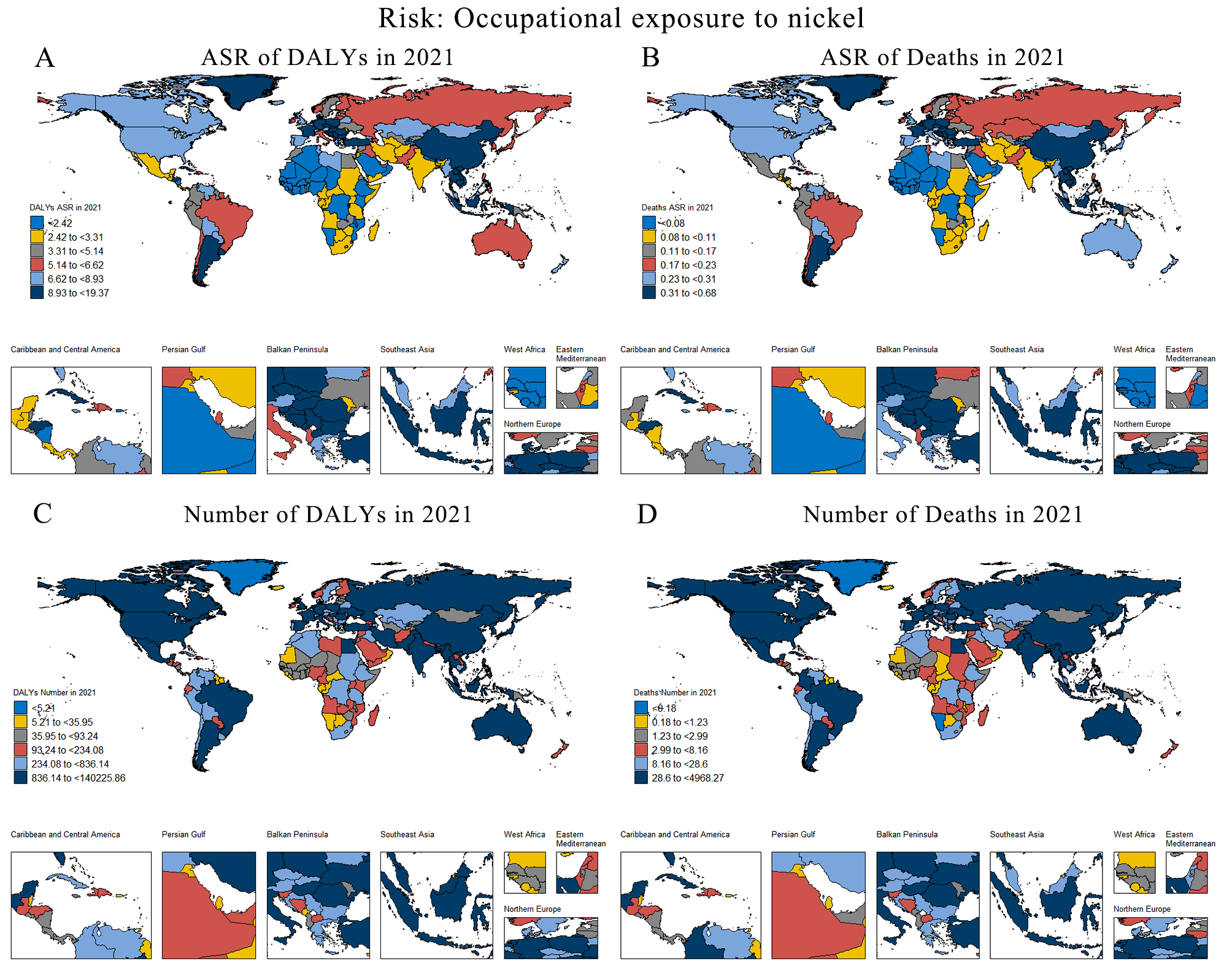


Figure S11 The heatmap for the global burden of nickel exposure in 2021. (A) ASR of DALYs.(B) ASR of Deaths.(C)Number of DALYs.(D)Number of Deaths.


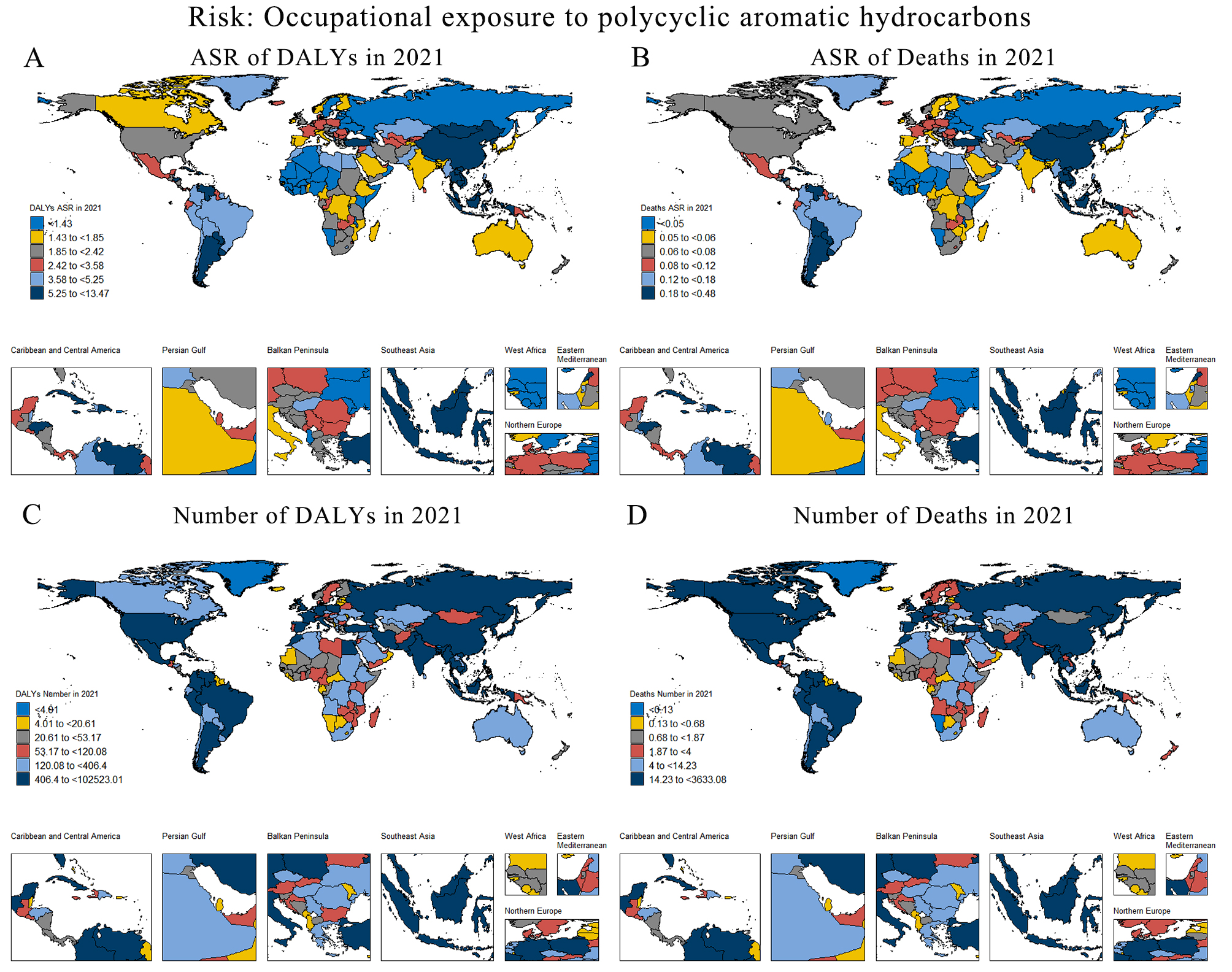


Figure S12 The heatmap for the global burden of polycyclic aromatic hydrocarbons exposure in 2021. (A) ASR of DALYs.(B) ASR of Deaths.(C)Number of DALYs.(D)Number of Deaths.


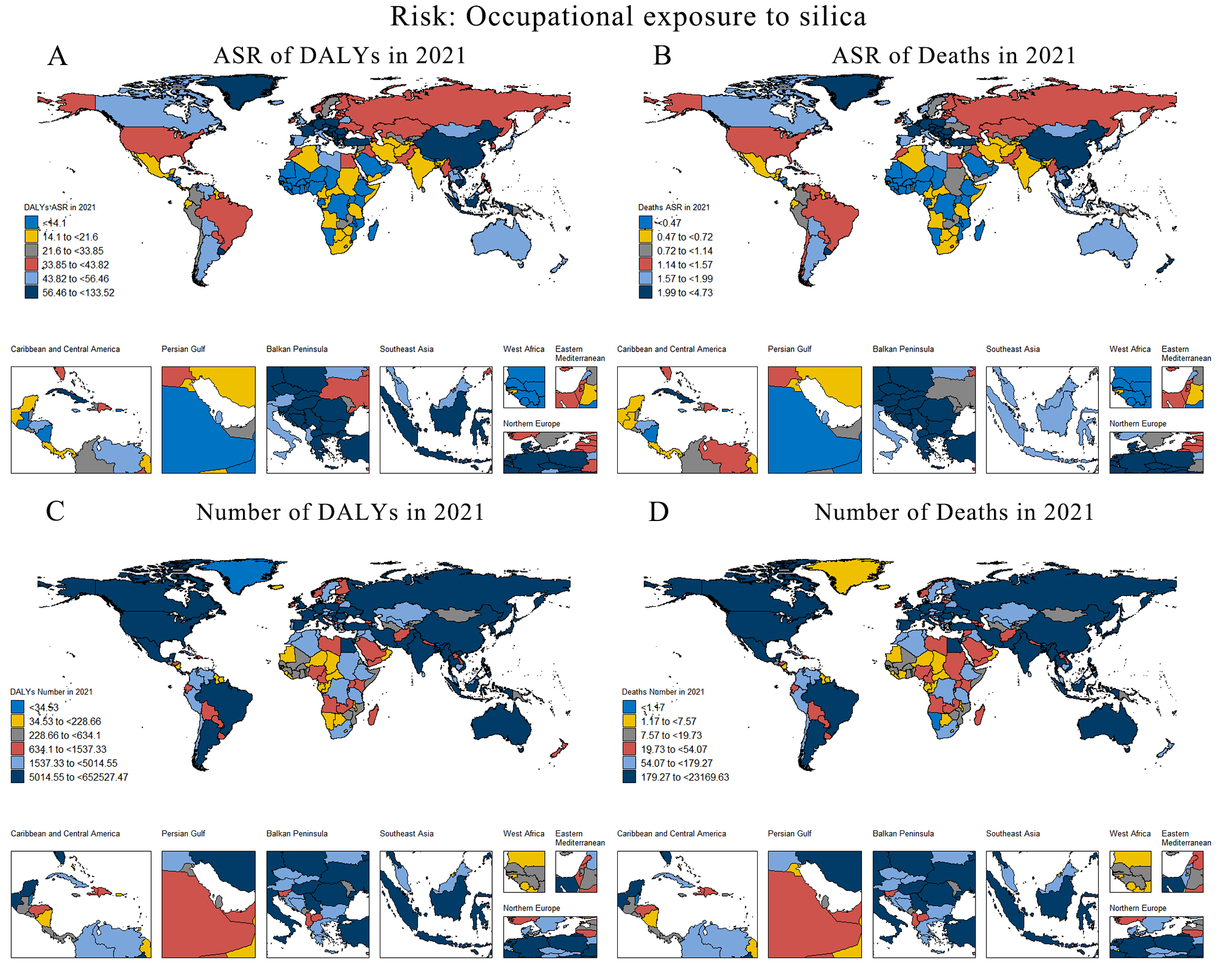


Figure S13 The heatmap for the global burden of silica exposure in 2021. (A) ASR of DALYs.(B) ASR of Deaths.(C)Number of DALYs.(D)Number of Deaths.


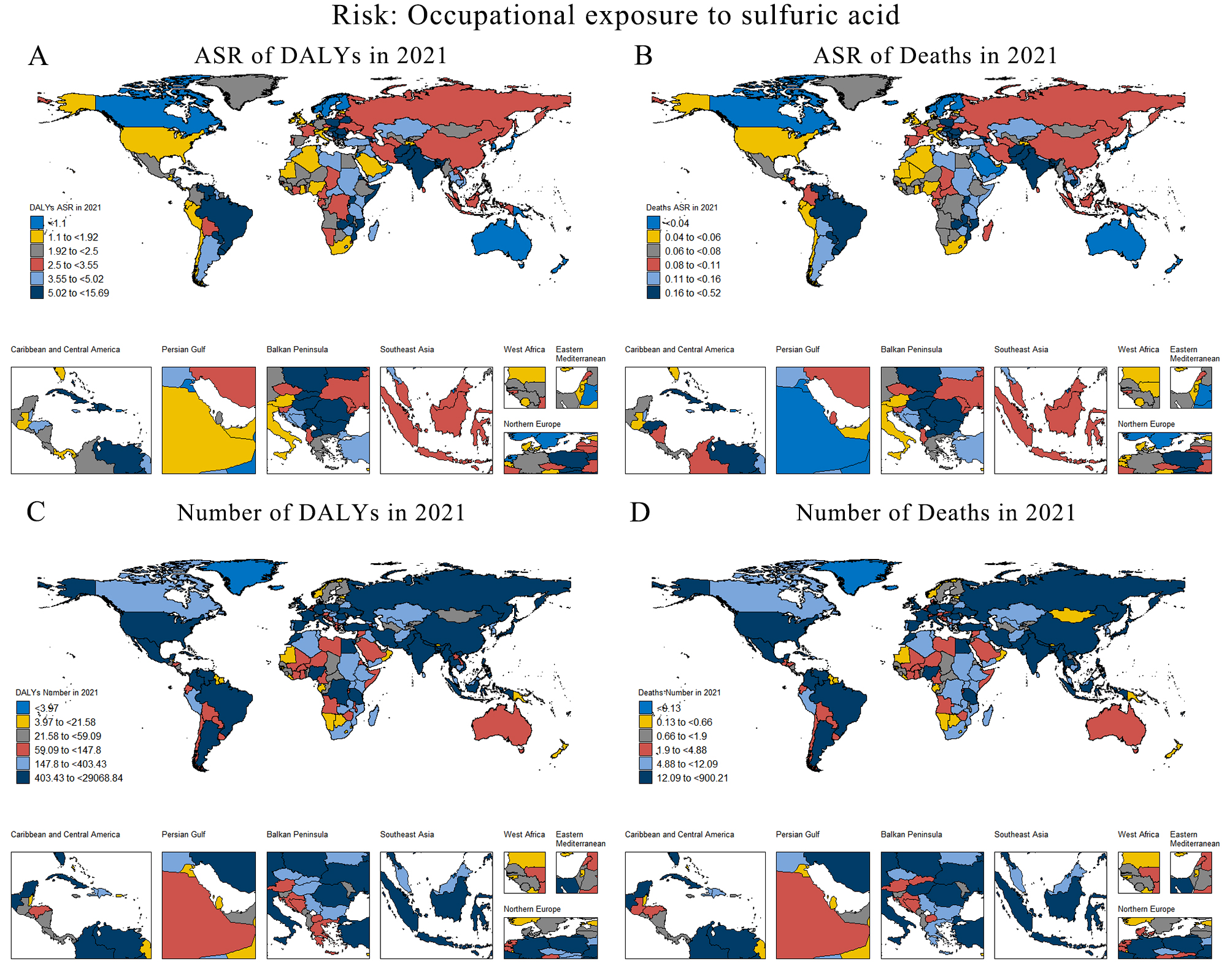


Figure S14 The heatmap for the global burden of sulfuric acid exposure in 2021. (A) ASR of DALYs.(B) ASR of Deaths.(C)Number of DALYs.(D)Number of Deaths.


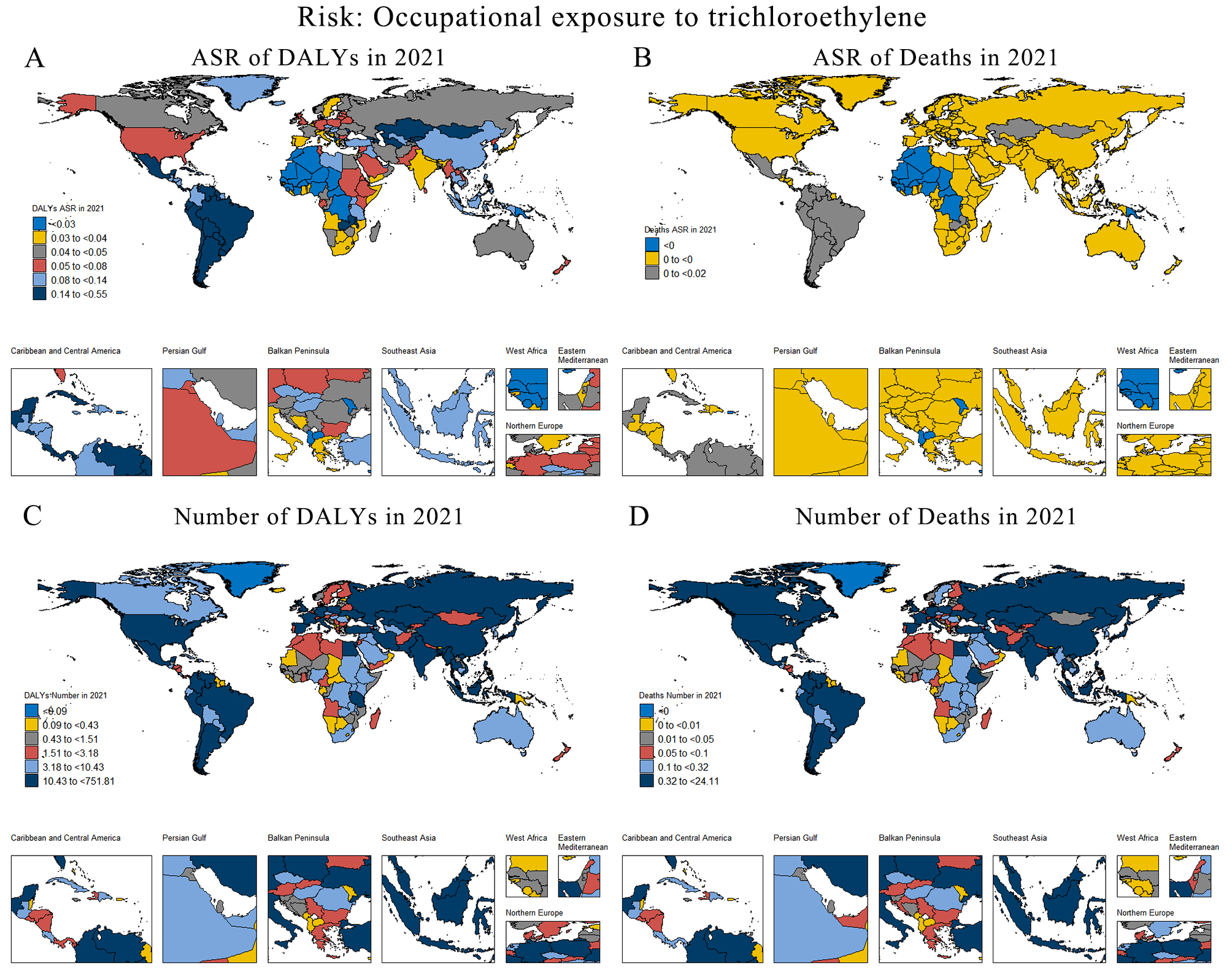


Figure S15 The heatmap for the global burden of trichloroethylene exposure in 2021. (A) ASR of DALYs.(B) ASR of Deaths.(C)Number of DALYs.(D)Number of Deaths.


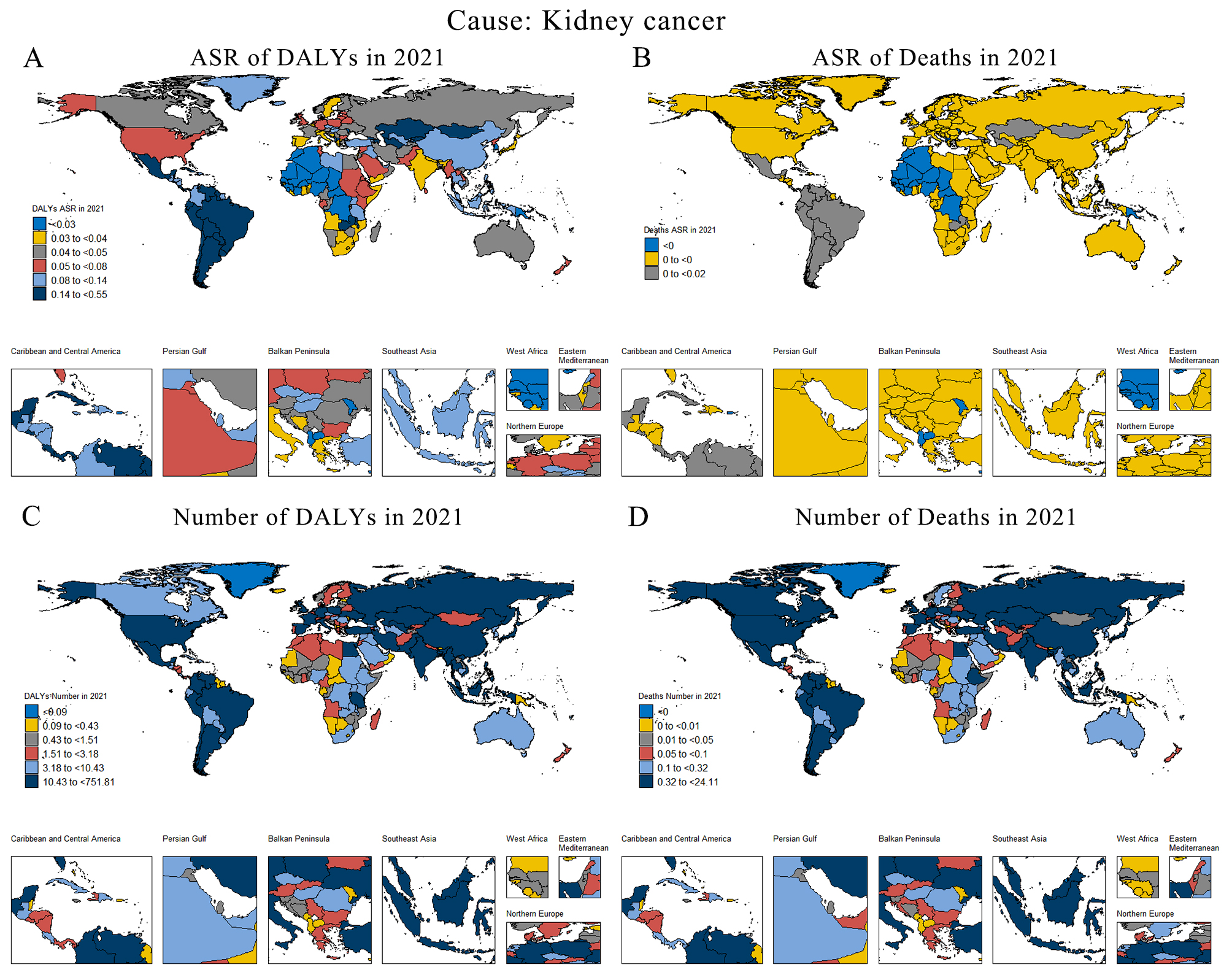


Figure S16 The heatmap for the global burden of kidney cancer in 2021. (A) ASR of DALYs.(B) ASR of Deaths.(C)Number of DALYs.(D)Number of Deaths.


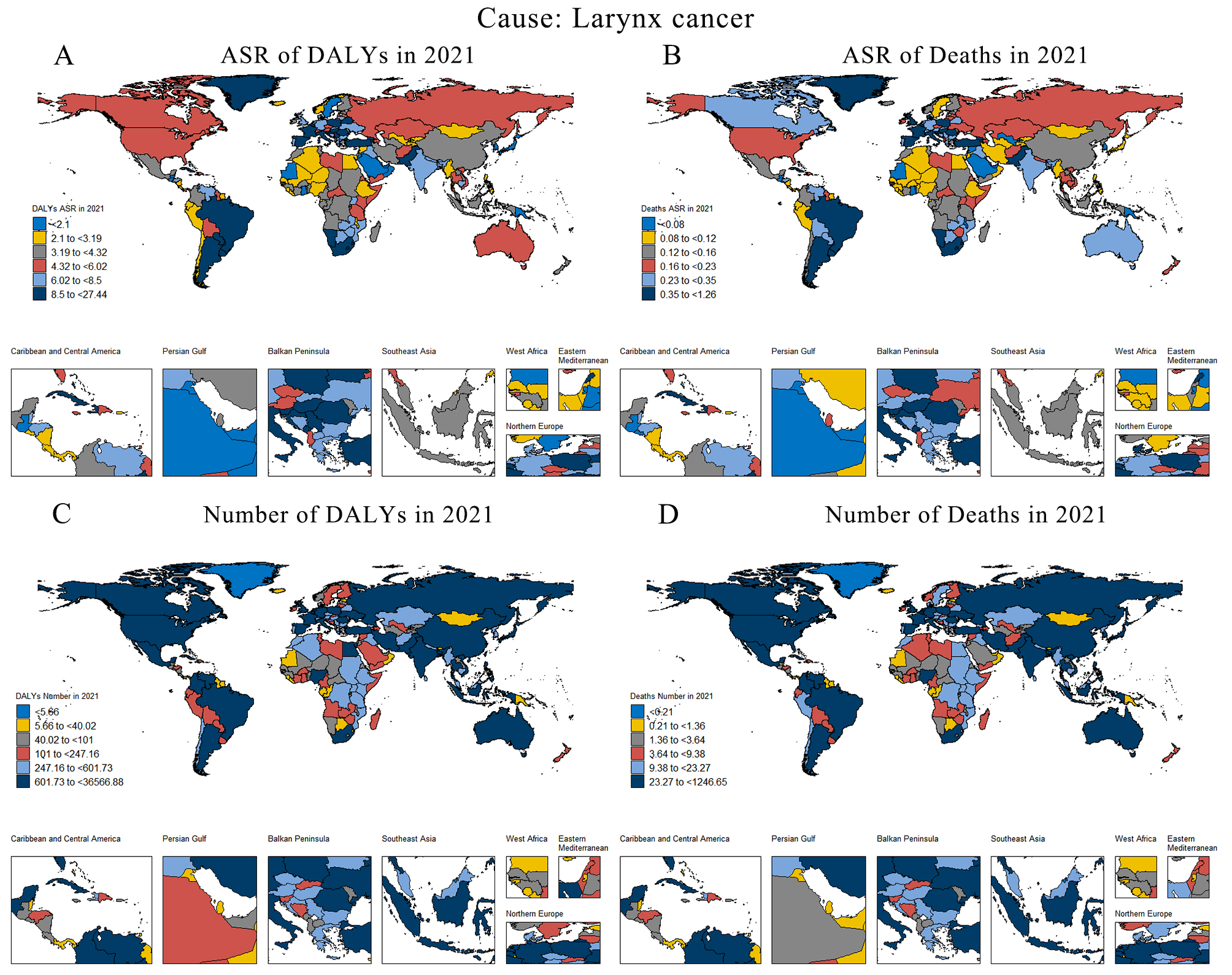


Figure S17 The heatmap for the global burden of larynx cancer in 2021. (A) ASR of DALYs.(B) ASR of Deaths.(C)Number of DALYs.(D)Number of Deaths.


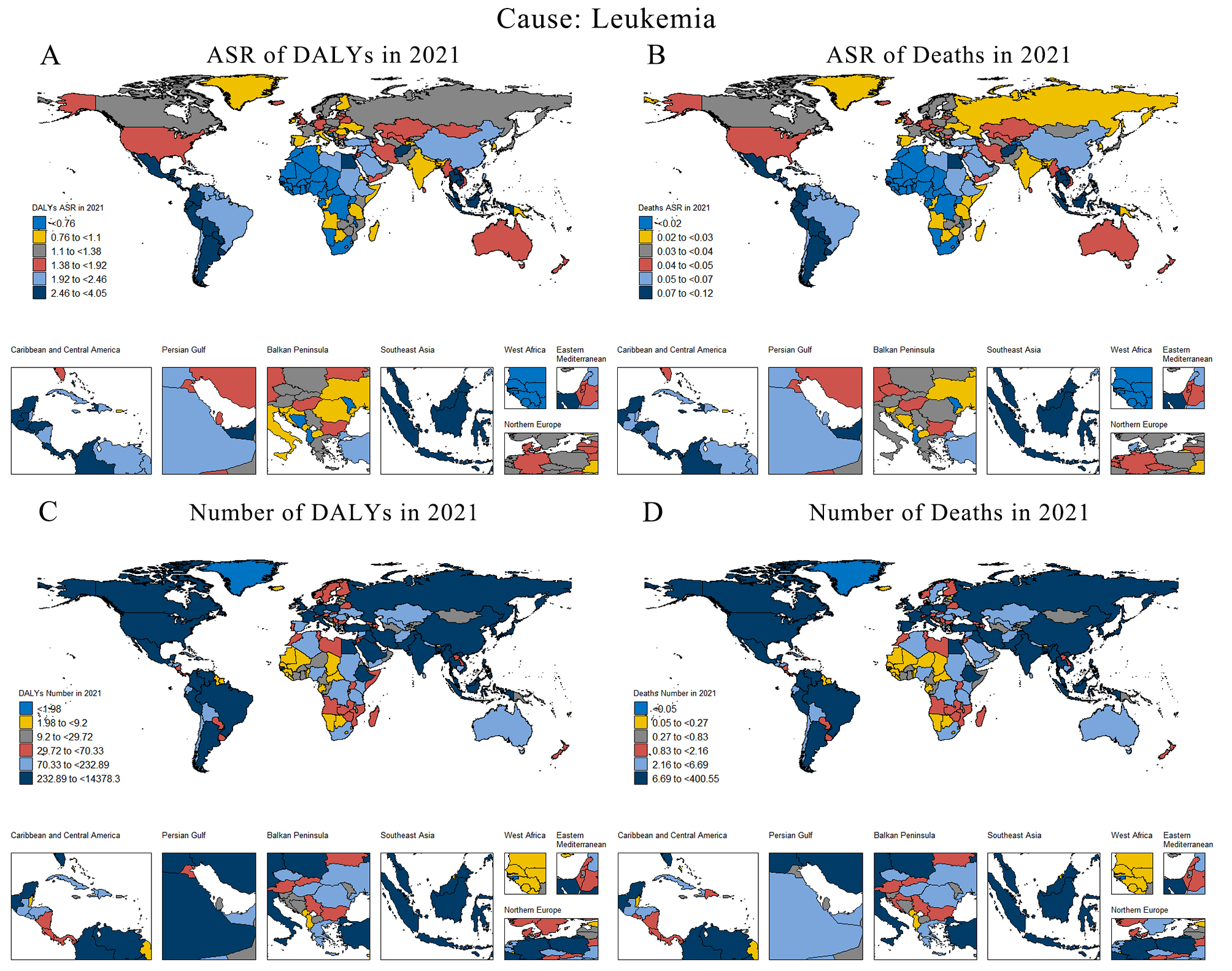


Figure S18 The heatmap for the global burden of leukemia in 2021. (A) ASR of DALYs.(B) ASR of Deaths.(C)Number of DALYs.(D)Number of Deaths.


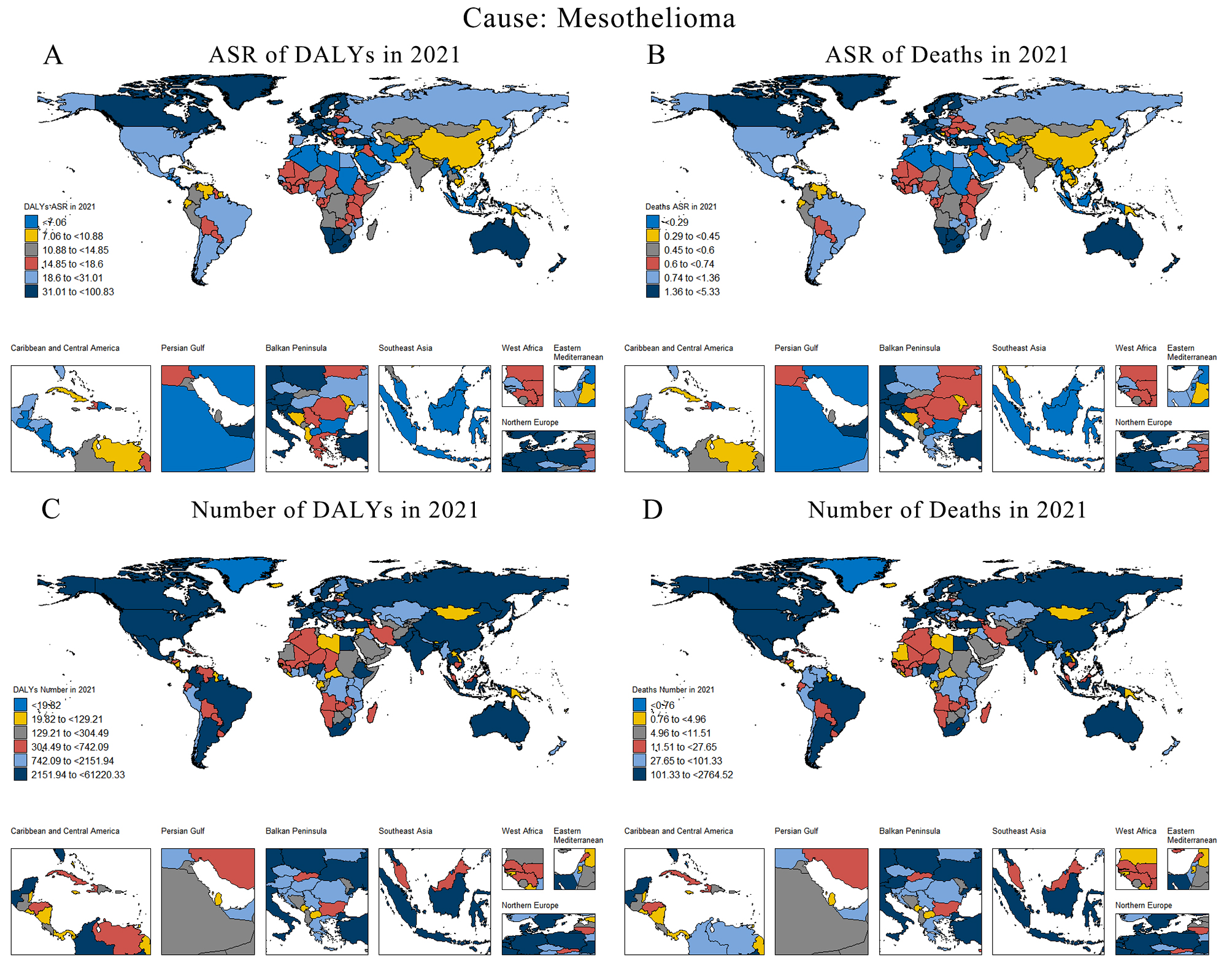


Figure S19 The heatmap for the global burden of mesothelioma in 2021. (A) ASR of DALYs.(B) ASR of Deaths.(C)Number of DALYs.(D)Number of Deaths.


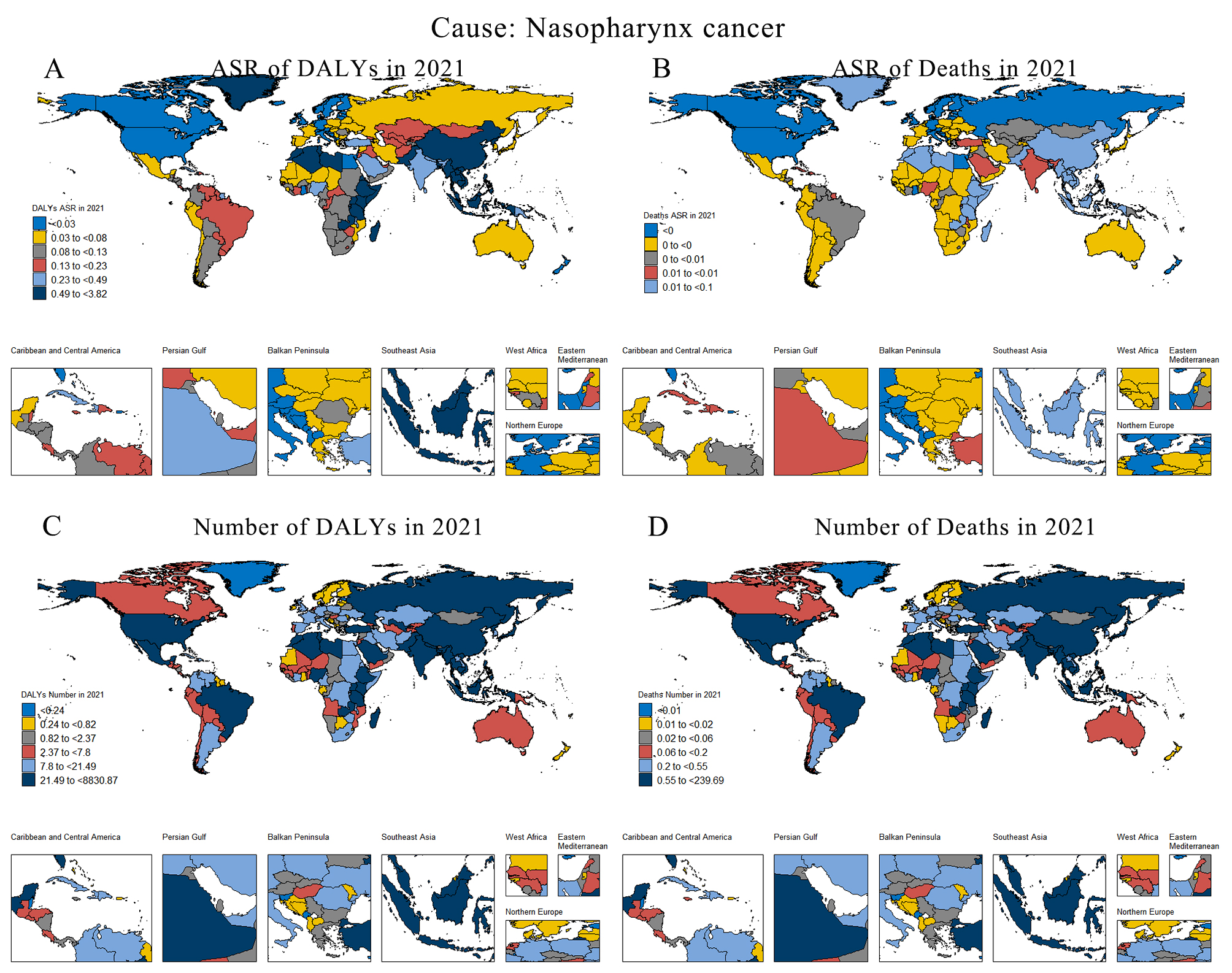


Figure S20 The heatmap for the global burden of nasopharynx cancer in 2021. (A) ASR of DALYs.(B) ASR of Deaths.(C)Number of DALYs.(D)Number of Deaths.


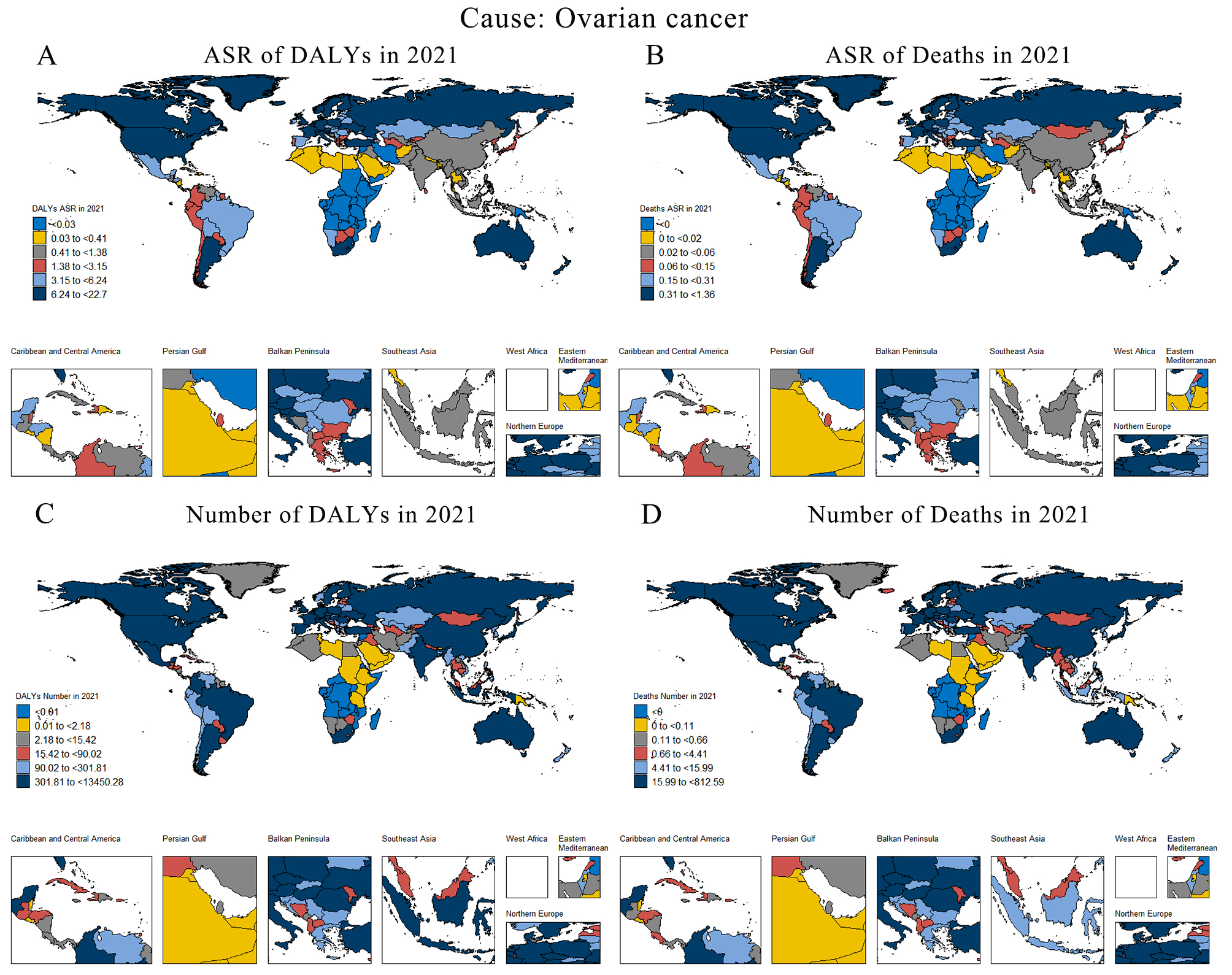


Figure S21 The heatmap for the global burden of ovarian cancer in 2021. (A) ASR of DALYs.(B) ASR of Deaths.(C)Number of DALYs.(D)Number of Deaths.


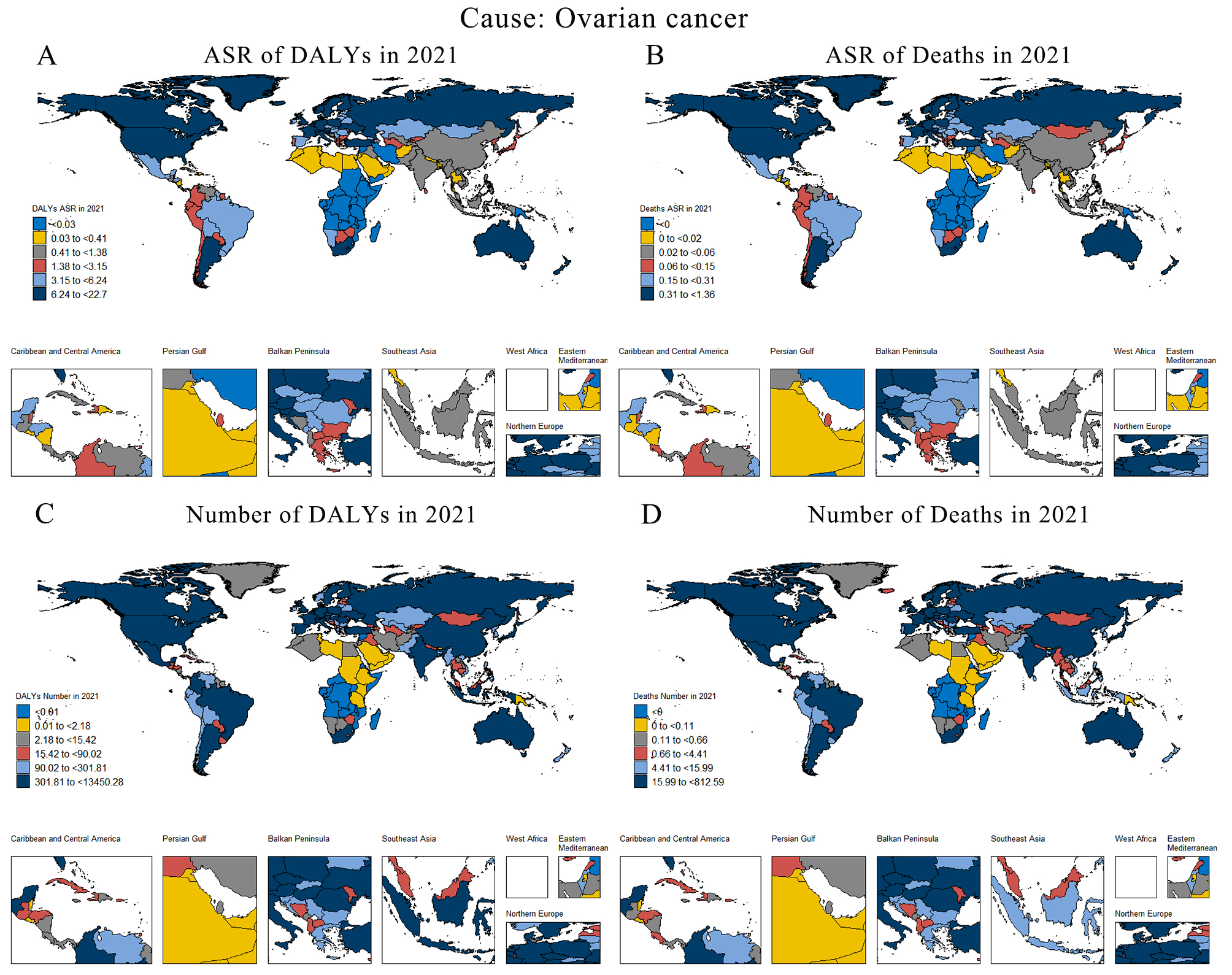


Figure S22 The heatmap for the global burden of tracheal bronchialus and lung cancer in 2021. (A) ASR of DALYs.(B) ASR of Deaths.(C)Number of DALYs.(D)Number of Deaths.

## Supplementary Tables

Table S1 Number and ASR of Deaths for Cancer in Individuals Aged 40 Years and Older Attributable to Occupational Carcinogen Exposure Risks in 2021, with Trends from 1990 to 2021 and Projections for 2030 and 2050

|  | 2021 | | 1990-2021 |
| --- | --- | --- | --- |
|  | Deaths cases | ASR-Deaths per 100,000 (95% UI) | EAPC of Deaths |
| Global | 325614.79 (244681.75 to 406667.12) | 11.45 (8.57 to 14.29) | -0.85 (-0.94 to -0.76) |
| Global Prediction of 2030 | 384192.41 (305387.10 to 462997.71) | 10.23 (8.13 to 12.34) |  |
| Global Prediction of 2050 | 516424.25 (8696.82 to 1090484.75) | 8.50 (0.13 to 18.25) |  |
| Sex |  |  |  |
| Male | 256947.29 (190656.86 to 325645.14) | 20.43 (15.05 to 25.92) | -1.16 (-1.26 to -1.06) |
| Female | 68667.51 (47613.52 to 89987.76) | 4.41 (3.06 to 5.78) | 0.26 (0.18 to 0.33) |
| Age (years) |  |  |  |
| 40-44 years | 3088.55 (2309.49 to 4011.63) | 0.62 (0.46 to 0.8) | -1.67 (-1.86 to -1.47) |
| 45-49 years | 6996.2 (4997.74 to 9316.15) | 1.48 (1.06 to 1.97) | -0.94 (-1.12 to -0.76) |
| 50-54 years | 15006.26 (10923.26 to 19739.53) | 3.37 (2.46 to 4.44) | -1.34 (-1.5 to -1.18) |
| 55-59 years | 25014.43 (18582.2 to 32199.3) | 6.32 (4.7 to 8.14) | -1.52 (-1.65 to -1.4) |
| 60-64 years | 34029.25 (26279.99 to 43518.12) | 10.63 (8.21 to 13.6) | -1.48 (-1.6 to -1.36) |
| 65-69 years | 47305.69 (36961.81 to 59416.3) | 17.15 (13.4 to 21.54) | -1.57 (-1.61 to -1.52) |
| 70-74 years | 55745.4 (43908.37 to 67535.14) | 27.08 (21.33 to 32.81) | -1.21 (-1.29 to -1.12) |
| 75-79 years | 50930.99 (39267.6 to 61743.73) | 38.62 (29.77 to 46.82) | -0.83 (-0.95 to -0.7) |
| 80-84 years | 42925.21 (31260.44 to 52852.04) | 49.01 (35.69 to 60.35) | -0.27 (-0.48 to -0.06) |
| 85-89 years | 29379.87 (20245.33 to 36750.32) | 64.26 (44.28 to 80.38) | 0.85 (0.65 to 1.05) |
| 90-94 years | 12102.92 (8120.93 to 15439.16) | 67.65 (45.4 to 86.3) | 1.42 (1.26 to 1.58) |
| 95+ years | 3090.02 (1824.59 to 4145.69) | 56.69 (33.48 to 76.06) | 1.79 (1.65 to 1.92) |
| SDI |  |  |  |
| High | 152995.3 (114343.61 to 187484.3) | 19.94 (14.97 to 24.45) | -1.19 (-1.3 to -1.08) |
| High-middle | 85767.96 (62759.6 to 111844.83) | 12.75 (9.31 to 16.64) | -0.56 (-0.66 to -0.45) |
| Middle | 3541.42 (2461.22 to 4891.91) | 7.42 (5.26 to 10.06) | 0.56 (0.44 to 0.68) |
| Low-middle | 16520.82 (12542.56 to 21356.83) | 3.43 (2.59 to 4.43) | 1.08 (1.05 to 1.11) |
| Low | 66474.45 (47428.87 to 89887.16) | 2.09 (1.44 to 2.92) | 0.22 (0.12 to 0.32) |
| GBD region |  |  |  |
| High-income | 168020.35 (125438.94 to 205811.14) | 20.71 (15.54 to 25.39) | -1.22 (-1.32 to -1.12) |
| Southeast Asia, East Asia, and Oceania | 93637.23 (64143.15 to 130869.35) | 9.77 (6.64 to 13.68) | 1.02 (0.83 to 1.21) |
| South Asia | 13464.37 (10014.54 to 17706.13) | 2.68 (1.99 to 3.53) | 0.62 (0.54 to 0.69) |
| Central Europe, Eastern Europe, and Central Asia | 21541.88 (15579.84 to 28147.57) | 9.62 (6.94 to 12.59) | -0.45 (-0.57 to -0.33) |
| North Africa and Middle East | 11642.73 (7661.43 to 16801.73) | 8.09 (5.24 to 11.79) | -1.09 (-1.37 to -0.81) |
| Latin America and Caribbean | 12309.6 (9425.04 to 15454.78) | 5.99 (4.57 to 7.53) | -0.37 (-0.44 to -0.3) |
| Sub-Saharan Africa | 4998.63 (3645.04 to 6722.16) | 3.36 (2.44 to 4.53) | -0.17 (-0.53 to 0.19) |
| Carcinogen risk |  |  |  |
| Arsenic | 10402.12 (2038.87 to 18387.14) | 0.35 (0.07 to 0.62) | -0.37 (-0.42 to -0.32) |
| Asbestos | 225327.78 (162880.23 to 285659.55) | 8.08 (5.83 to 10.24) | -1.08 (-1.2 to -0.96) |
| Benzene | 1030.64 (309.43 to 1708.73) | 0.04 (0.01 to 0.06) | -0.16 (-0.22 to -0.09) |
| Beryllium | 303.88 (175.16 to 451.65) | 0.01 (0.01 to 0.02) | 0.16 (0.07 to 0.24) |
| Cadmium | 797.6 (479.72 to 1150.6) | 0.03 (0.02 to 0.04) | 0.58 (0.5 to 0.67) |
| Chromium | 1727.05 (1240.82 to 2303.59) | 0.06 (0.04 to 0.08) | 0.82 (0.74 to 0.9) |
| Diesel engine exhaust | 21307.73 (14817.24 to 28640.34) | 0.72 (0.5 to 0.96) | 0.82 (0.77 to 0.87) |
| Formaldehyde | 778.31 (377.33 to 1346.84) | 0.03 (0.01 to 0.05) | -0.87 (-0.98 to -0.76) |
| Nickel | 9728.25 (739.09 to 25359.33) | 0.33 (0.02 to 0.85) | -0.38 (-0.42 to -0.33) |
| Polycyclic aromatic hydrocarbons | 5974.03 (3735.89 to 8458.4) | 0.2 (0.13 to 0.28) | 0.79 (0.71 to 0.87) |
| Silica | 53285.63 (23311.69 to 84409.26) | 1.79 (0.78 to 2.84) | -0.54 (-0.57 to -0.51) |
| Sulfuric acid | 3556.03 (1479.5 to 6404.82) | 0.12 (0.05 to 0.22) | -1.56 (-1.63 to -1.49) |
| Trichloroethylene | 77.03 (16.92 to 141.89) | 0 (0 to 0) | 1.27 (1.19 to 1.34) |
| Cancer |  |  |  |
| Kidney cancer | 77.03 (16.92 to 141.89) | 0 (0 to 0) | 1.27 (1.19 to 1.34) |
| Larynx cancer | 6889.14 (3935.59 to 10655.47) | 0.24 (0.14 to 0.37) | -1.8 (-1.86 to -1.74) |
| Leukemia | 1357.66 (622.75 to 2076.79) | 0.05 (0.02 to 0.07) | -0.1 (-0.17 to -0.03) |
| Mesothelioma | 26974.82 (24031.37 to 29419.12) | 0.95 (0.85 to 1.04) | -0.18 (-0.31 to -0.06) |
| Nasopharynx cancer | 447.72 (92.28 to 998.29) | 0.02 (0 to 0.03) | -1.47 (-1.66 to -1.28) |
| Ovarian cancer | 5583.93 (2576.29 to 9027.9) | 0.2 (0.09 to 0.33) | -1.32 (-1.45 to -1.19) |
| Tracheal bronchialus and lung cancer | 284284.49 (205777.45 to 363339.6) | 9.99 (7.21 to 12.76) | -0.88 (-0.97 to -0.79) |

Table S2 Decomposition of aging, population, and epidemiological change for Cancer in Individuals Aged 40 Years and Older Attributable to Occupational Carcinogen Exposure Risks from 1990 to 2021

| Measure | Location | Overall difference | Aging | Population | Epidemiological change | percent change of Aging | percent change of Population | percent change of Epidemiological change |
| --- | --- | --- | --- | --- | --- | --- | --- | --- |
| DALYs | Global | 2398563.23 | 390412.42 | 4252725.55 | -2244574.74 | 16.28 | 177.30 | -93.58 |
|  | High SDI | 326284.10 | 335387.94 | 1318592.70 | -1327696.54 | 102.79 | 404.12 | -406.91 |
|  | High-middle SDI | 626072.36 | 81943.79 | 1034392.42 | -490263.85 | 13.09 | 165.22 | -78.31 |
|  | Middle SDI | 1077026.87 | 77167.65 | 931683.34 | 68175.88 | 7.16 | 86.51 | 6.33 |
|  | Low-middle SDI | 308181.30 | 5174.41 | 226067.50 | 76939.38 | 1.68 | 73.36 | 24.97 |
|  | Low SDI | 58570.41 | -3085.14 | 58747.24 | 2908.32 | -5.27 | 100.30 | 4.97 |
|  | High-income | 218710.76 | 379130.25 | 1310510.21 | -1470929.70 | 173.35 | 599.20 | -672.55 |
|  | Southeast Asia, East Asia, and Oceania | 1564528.76 | 127116.84 | 1190707.84 | 246704.08 | 8.12 | 76.11 | 15.77 |
|  | South Asia | 249924.62 | 8264.38 | 198985.34 | 42674.89 | 3.31 | 79.62 | 17.08 |
|  | Central Europe, Eastern Europe, and Central Asia | -7507.58 | 3017.83 | 133204.49 | -143729.89 | -40.20 | -1774.27 | 1914.46 |
|  | North Africa and Middle East | 131501.34 | -10358.97 | 248182.17 | -106321.87 | -7.88 | 188.73 | -80.85 |
|  | Latin America and Caribbean | 168621.15 | 15969.94 | 191516.59 | -38865.38 | 9.47 | 113.58 | -23.05 |
|  | Sub-Saharan Africa | 72784.19 | -6420.21 | 85286.83 | -6082.44 | -8.82 | 117.18 | -8.36 |
| Deaths | Global | 131370.09 | 27981.34 | 188411.64 | -85022.88 | 21.30 | 143.42 | -64.72 |
|  | High SDI | 38416.65 | 23018.37 | 66611.19 | -51212.91 | 59.92 | 173.39 | -133.31 |
|  | High-middle SDI | 34463.86 | 6204.47 | 42384.04 | -14124.65 | 18.00 | 122.98 | -40.98 |
|  | Middle SDI | 44965.86 | 4949.94 | 34666.40 | 5349.51 | 11.01 | 77.09 | 11.90 |
|  | Low-middle SDI | 11331.91 | 454.87 | 7951.12 | 2925.92 | 4.01 | 70.17 | 25.82 |
|  | Low SDI | 2061.77 | -111.57 | 2028.84 | 144.50 | -5.41 | 98.40 | 7.01 |
|  | High-income | 37507.25 | 27151.51 | 66613.50 | -56257.76 | 72.39 | 177.60 | -149.99 |
|  | Southeast Asia, East Asia, and Oceania | 65895.85 | 8079.46 | 44523.53 | 13292.87 | 12.26 | 67.57 | 20.17 |
|  | South Asia | 9208.19 | 618.07 | 6881.63 | 1708.50 | 6.71 | 74.73 | 18.55 |
|  | Central Europe, Eastern Europe, and Central Asia | 2999.51 | 759.04 | 5060.55 | -2820.09 | 25.31 | 168.71 | -94.02 |
|  | North Africa and Middle East | 5505.41 | -365.40 | 9460.34 | -3589.54 | -6.64 | 171.84 | -65.20 |
|  | Latin America and Caribbean | 7489.27 | 1050.33 | 7657.94 | -1219.00 | 14.02 | 102.25 | -16.28 |
|  | Sub-Saharan Africa | 2764.61 | -311.67 | 3132.52 | -56.24 | -11.27 | 113.31 | -2.03 |

Table S3 Slope indices of ASRs for Cancer in Individuals Aged 40 Years and Older Attributable to Occupational Carcinogen Exposure Risks from 1990 to 2021

| Location | Year | Measure | Slope Indicies | | |
| --- | --- | --- | --- | --- | --- |
|  |  |  | lm (95%CI) | ncvTest p-value | rlm (95%CI) |
| Global | 1990 | Deaths | 25.22(20.94 to 29.51) | <0.001 | 16.21(13.71 to 18.71) |
|  |  | DALYs | 558.72(467.31 to 650.13) | <0.001 | 387.43(331.01 to 443.86) |
|  | 2021 | Deaths | 15.46(12.71 to 18.2) | <0.001 | 11.66(9.9 to 13.42) |
|  |  | DALYs | 299.59(244.86 to 354.32) | <0.001 | 240.97(204.1 to 277.84) |
| High SDI | 1990 | Deaths | 23.2(9.18 to 37.23) | 0.9235 | 23.41(9.92 to 36.9) |
|  |  | DALYs | 479.13(184.45 to 773.8) | 0.7529 | 485.64(208.41 to 762.86) |
|  | 2021 | Deaths | 9.46(-0.24 to 19.17) | 0.7382 | 9.72(0.52 to 18.93) |
|  |  | DALYs | 154.27(-36.83 to 345.38) | 0.7683 | 157.65(-19.23 to 334.53) |
| High-middle SDI | 1990 | Deaths | 13.17(-1.46 to 27.8) | 0.2889 | 10.77(1.24 to 20.3) |
|  |  | DALYs | 287.8(-48.4 to 623.99) | 0.5450 | 248.64(27.74 to 469.55) |
|  | 2021 | Deaths | -3.34(-11.81 to 5.14) | 0.3411 | -3.52(-10.41 to 3.37) |
|  |  | DALYs | -96.37(-272.51 to 79.77) | 0.1209 | -96.97(-242.86 to 48.93) |
| Middle SDI | 1990 | Deaths | 2.69(-0.08 to 5.47) | 0.0162 | 1.78(-0.41 to 3.96) |
|  |  | DALYs | 69.68(0.79 to 138.56) | 0.0046 | 44.29(-9.93 to 98.51) |
|  | 2021 | Deaths | 0.38(-2.27 to 3.03) | 0.0179 | -0.59(-2.68 to 1.5) |
|  |  | DALYs | 3.79(-56.71 to 64.3) | 0.0363 | -15.31(-63.95 to 33.33) |
| Low-middle SDI | 1990 | Deaths | 2.24(0.41 to 4.07) | 0.0258 | 1.91(0.23 to 3.59) |
|  |  | DALYs | 54.42(8.78 to 100.06) | 0.0438 | 47.99(1.01 to 94.97) |
|  | 2021 | Deaths | 2.16(-0.81 to 5.13) | 0.4230 | 2.44(0.58 to 4.3) |
|  |  | DALYs | 48.86(-23.94 to 121.66) | 0.2906 | 58.3(13.41 to 103.19) |
| Low SDI | 1990 | Deaths | 1.52(0.58 to 2.46) | 0.0029 | 1.14(0.52 to 1.76) |
|  |  | DALYs | 38.39(14.53 to 62.26) | 0.0154 | 30.68(14.51 to 46.84) |
|  | 2021 | Deaths | 1.33(0.32 to 2.34) | <0.001 | 0.75(0.02 to 1.48) |
|  |  | DALYs | 33.6(7.56 to 59.63) | <0.001 | 20.32(0.57 to 40.06) |

Table S4 Intercept indicies of ASR for Cancer in Individuals Aged 40 Years and Older Attributable to Occupational Carcinogen Exposure Risks from 1990 to 2021

| Location | Year | Measure | Intercept Indicies | | |
| --- | --- | --- | --- | --- | --- |
|  |  |  | lm (95%CI) | ncvTest p-value | rlm (95%CI) |
| Global | 1990 | Deaths | -1.5(-3.87 to 0.86) | <0.001 | 0.45(-0.93 to 1.83) |
|  |  | DALYs | -18.3(-68.79 to 32.19) | <0.001 | 18.58(-12.58 to 49.75) |
|  | 2021 | Deaths | 1.26(-0.29 to 2.82) | <0.001 | 1.79(0.79 to 2.78) |
|  |  | DALYs | 49.55(18.51 to 80.59) | <0.001 | 54.54(33.63 to 75.45) |
| High SDI | 1990 | Deaths | 18.14(11.48 to 24.8) | 0.9235 | 16.56(10.15 to 22.96) |
|  |  | DALYs | 415.97(276.04 to 555.9) | 0.7529 | 381.51(249.87 to 513.15) |
|  | 2021 | Deaths | 14.52(9.2 to 19.84) | 0.7382 | 13.49(8.44 to 18.54) |
|  |  | DALYs | 306.5(201.76 to 411.23) | 0.7683 | 284.72(187.78 to 381.66) |
| High-middle SDI | 1990 | Deaths | 1.85(-8.97 to 12.68) | 0.2889 | 2.39(-4.66 to 9.44) |
|  |  | DALYs | 71.95(-176.68 to 320.59) | 0.5450 | 72.44(-90.93 to 235.81) |
|  | 2021 | Deaths | 12.34(5.59 to 19.1) | 0.3411 | 11.76(6.27 to 17.24) |
|  |  | DALYs | 301.96(161.66 to 442.26) | 0.1209 | 290.17(173.97 to 406.38) |
| Middle SDI | 1990 | Deaths | 4.07(2.27 to 5.87) | 0.0162 | 4.05(2.63 to 5.47) |
|  |  | DALYs | 102.49(57.74 to 147.25) | 0.0046 | 104.42(69.2 to 139.65) |
|  | 2021 | Deaths | 5.05(3.35 to 6.74) | 0.0179 | 5.17(3.83 to 6.5) |
|  |  | DALYs | 127.09(88.5 to 165.68) | 0.0363 | 127.86(96.84 to 158.89) |
| Low-middle SDI | 1990 | Deaths | 2.43(1.08 to 3.78) | 0.0258 | 2.46(1.21 to 3.7) |
|  |  | DALYs | 65.61(31.8 to 99.41) | 0.0438 | 65.19(30.39 to 99.98) |
|  | 2021 | Deaths | 3.71(2.07 to 5.34) | 0.4230 | 3.09(2.06 to 4.11) |
|  |  | DALYs | 96(55.88 to 136.12) | 0.2906 | 78.5(53.76 to 103.24) |
| Low SDI | 1990 | Deaths | 1.51(0.94 to 2.08) | 0.0029 | 1.6(1.22 to 1.97) |
|  |  | DALYs | 41.11(26.63 to 55.59) | 0.0154 | 42.38(32.57 to 52.19) |
|  | 2021 | Deaths | 1.86(1.31 to 2.42) | <0.001 | 2(1.6 to 2.4) |
|  |  | DALYs | 49.23(34.98 to 63.48) | <0.001 | 52.2(41.4 to 63.01) |

Table S5 Concentration indicies of ASR for Cancer in Individuals Aged 40 Years and Older Attributable to Occupational Carcinogen Exposure Risks from 1990 to 2021

| Location | Year | Measure | Concentration Indicies | | |
| --- | --- | --- | --- | --- | --- |
|  |  |  | concentration Indicies (95%CI) | SE | p-value |
| Global | 1990 | Deaths | 0.3974 (0.3445 to 0.4504) | 0.0270 | <0.001 |
|  |  | DALYs | 0.2897 (0.2525 to 0.3269) | 0.0190 | <0.001 |
|  | 2021 | Deaths | 0.4334 (0.3755 to 0.4914) | 0.0296 | <0.001 |
|  |  | DALYs | 0.3419 (0.3005 to 0.3833) | 0.0211 | <0.001 |
| High SDI | 1990 | Deaths | -0.0123 (-0.0996 to 0.0751) | 0.0446 | 0.7847 |
|  |  | DALYs | -0.0571 (-0.1308 to 0.0166) | 0.0376 | 0.1369 |
|  | 2021 | Deaths | -0.0017 (-0.0905 to 0.087) | 0.0453 | 0.9695 |
|  |  | DALYs | -0.0406 (-0.112 to 0.0307) | 0.0364 | 0.2710 |
| High-middle SDI | 1990 | Deaths | 0.1971 (0.1129 to 0.2813) | 0.0430 | <0.001 |
|  |  | DALYs | -0.0297 (-0.073 to 0.0136) | 0.0221 | 0.1855 |
|  | 2021 | Deaths | 0.2095 (0.1122 to 0.3068) | 0.0497 | <0.001 |
|  |  | DALYs | -0.0171 (-0.0701 to 0.0359) | 0.0271 | 0.5306 |
| Middle SDI | 1990 | Deaths | 0.1394 (0.0667 to 0.2122) | 0.0371 | 0.0006 |
|  |  | DALYs | -0.0573 (-0.1276 to 0.0129) | 0.0358 | 0.1176 |
|  | 2021 | Deaths | 0.16 (0.0825 to 0.2376) | 0.0396 | <0.001 |
|  |  | DALYs | -0.0472 (-0.1248 to 0.0303) | 0.0396 | 0.2396 |
| Low-middle SDI | 1990 | Deaths | 0.0293 (-0.0372 to 0.0958) | 0.0339 | 0.3926 |
|  |  | DALYs | -0.005 (-0.0757 to 0.0657) | 0.0361 | 0.8904 |
|  | 2021 | Deaths | 0.0244 (-0.0452 to 0.0939) | 0.0355 | 0.4959 |
|  |  | DALYs | -0.006 (-0.0788 to 0.0668) | 0.0371 | 0.8725 |
| Low SDI | 1990 | Deaths | 0.0392 (-0.0245 to 0.103) | 0.0325 | 0.2366 |
|  |  | DALYs | 0.0593 (0.006 to 0.1125) | 0.0272 | 0.0366 |
|  | 2021 | Deaths | 0.0435 (-0.0224 to 0.1094) | 0.0336 | 0.2053 |
|  |  | DALYs | 0.0589 (6e-04 to 0.1172) | 0.0297 | 0.0564 |
